# Species sorting and mass effect along forest succession: Evidence from taxonomic, functional, and phylogenetic diversity of amphibian communities

**DOI:** 10.1002/ece3.5110

**Published:** 2019-04-08

**Authors:** Omar Hernández‐Ordóñez, Bráulio A. Santos, Robert Alexander Pyron, Víctor Arroyo‐Rodríguez, J. Nicolás Urbina‐Cardona, Miguel Martínez‐Ramos, Gabriela Parra‐Olea, Víctor Hugo Reynoso

**Affiliations:** ^1^ Departamento de Zoología, Instituto de Biología Universidad Nacional Autónoma de México Ciudad de México México; ^2^ Departamento de Sistemática e Ecologia Universidade Federal da Paraíba João Pessoa, Paraíba Brazil; ^3^ Department of Biological Sciences The George Washington University Washington District of Columbia; ^4^ Instituto de Investigaciones en Ecosistemas y Sustentabilidad Universidad Nacional Autónoma de México Morelia México; ^5^ Ecology and Territory Department, School of Rural and Environmental Studies Pontificia Universidad Javeriana Bogota Colombia

**Keywords:** anuran, caecilian, environmental filtering, metacommunity, salamander, secondary forest

## Abstract

1. Species recovery after forest disturbance is a highly studied topic in the tropics, but considerable debate remains on the role of secondary forests as biodiversity repositories, especially regarding the functional and phylogenetic dimensions of biodiversity. Also, studies generally overlook how alpha and beta diversities interact to produce gamma diversity along successional gradients.
2. We used a metacommunity approach to assess how species sorting (i.e., environmental filtering) and mass effect (i.e., source‐sink dynamics) affect 14 complementary metrics of amphibian taxonomic, functional, and phylogenetic diversity along a successional gradient in southern Mexico. As amphibians have narrow environmental tolerances and low dispersal capabilities, we expected that species sorting may be relatively more important than mass effect in structuring amphibian communities.
3. Between 2010 and 2012, we sampled frogs, salamanders, and caecilians in 23 communities distributed in four successional stages: young (2–5 years old) and intermediate (13–28 years old) secondary forests, old‐growth forest fragments, and old‐growth continuous forest. We assessed 15 ecologically relevant functional traits per species and used a time‐calibrated molecular phylogeny.
4. We recorded 1,672 individuals belonging to 30 species and 11 families. Supporting our expectations from the species sorting perspective, from the poorest (younger forests) to the best quality (continuous forest) scenarios, we observed (a) an increase in alpha diversity regardless of species abundances; (b) a clear taxonomic segregation across successional stages; (c) an increase in functional richness and dispersion; (d) an increase in mean phylogenetic distance and nearest taxon index; and (e) a reduction in mean nearest taxon distance. However, 10 species occurred in all successional stages, resulting in relatively low beta diversity. This supports a mass effect, where interpatch migrations contribute to prevent local extinctions and increase compositional similarity at the regional scale.
5. Our findings indicate that amphibian metacommunities along forest successional gradients are mainly structured by species sorting, but mass effects may also play a role if high levels of forest cover are conserved in the region. In fact, secondary forests and forest fragments can potentially safeguard different aspects of amphibian diversity, but their long‐term conservation value requires preventing additional deforestation.

## 1 INTRODUCTION

Forest conversion to agricultural fields has been the fate of many tropical rainforests worldwide (Lewis, Edwards, & Galbraith, 2015; Qin et al., 2017). This global phenomenon generates numerous benefits to society such as food, medicine, and wood, but often this conversion occurs in detriment to other ecosystem functions that are also beneficial to humans, such as water supply and carbon sequestration (FAO, 2016). Because many converted areas become unproductive a few years after a forest is cleared, abandoned fields at varying successional stages are increasingly found in human‐modified tropical landscapes (Bowen, McAlpine, House, & Smith, 2007; Chazdon, 2003). However, after the abandonment of agricultural and livestock fields, the process of secondary succession takes place and fauna colonize secondary forests (Chazdon et al., 2009; Dunn, 2004). The conservation value of these secondary forests remains poorly understood since most studies usually ignore the functional and phylogenetic dimensions of biodiversity, and we know very little about how diversity is distributed across space and time (Arroyo‐Rodríguez et al., 2017; Barlow et al., 2016). This limits our understanding of how degraded forests function (Guariguata & Ostertag, 2001). It also generates uncertainty in restoration and conservation programs (Melo, Arroyo‐Rodríguez, Fahrig, Martínez‐Ramos, & Tabarelli, 2013) and fuels the debate on the role of secondary forests as biodiversity repositories (Arroyo‐Rodríguez et al., 2017).

There are a large number of studies showing that species diversity increases along successional gradients (Chazdon, 2014; Dent & Wright, 2009). However, traditional approaches are based on measuring entropy rather than on measures of true diversity (Jost, 2006), and thus, they cannot be used to decouple gamma diversity into its independent alpha and beta components (Jost, 2007). Even if metrics of true diversity are used, a taxonomic assessment is not enough to estimate community's total diversity, since co‐occurring individuals often do not belong to a single lineage or play the same ecological role (Cavender‐Bares, Kozak, Fine, & Kembel, 2009; Pausas & Verdu, 2010). Thus, taxonomic diversity metrics need to be complemented with functional and phylogenetic metrics to better understand changes in alpha, beta, and gamma diversities across time and space (Jarzyna & Jetz, 2016).

Identifying the mechanisms underlying the changes in diversity along environmental gradients is critical to determine the successional pathways of degrading and regenerating tropical forests (Arroyo‐Rodríguez et al., 2017). The four major perspectives of the metacommunity approach (Leibold et al., 2004), namely, the patch dynamic, species sorting, mass effect, and neutral perspectives, provide a good theoretical framework for determining these mechanisms. The patch dynamic perspective is less likely to explain changes along successional gradients as it assumes that patches are identical and abandoned fields and degraded forest remnants usually violate this assumption. The neutral perspective assumes that all individuals are ecologically equivalent along the gradient, which is also not likely in human‐modified tropical landscapes where early successional species tend to proliferate (winners), and late successional species tend to disappear (losers; Suazo‐Ortuño et al., 2018; Tabarelli, Peres, & Melo, 2012). Therefore, species sorting, mass effect, or both probably structure the metacommunity along a successional gradient.

From the species sorting perspective, local patches are heterogeneous, species are separated into spatial niches, and dispersal is not sufficient to alter their distribution (Leibold et al., 2004). This may produce communities that are distinct locally and regionally, resulting in clear differentiation across successional stages in both the alpha and beta diversity components. From the mass effect perspective, the effect of immigration and emigration on local population dynamics is more important than patch quality (Leibold et al., 2004). In such a system, species can be rescued from local competitive exclusion in communities where they are bad competitors by immigration from communities where they are strong competitors (i.e., source‐sink relations; Leibold et al., 2004). This regional compensation of local competitive abilities may decrease β‐diversity at the regional scale (Mouquet & Loreau, 2002; Pardini, Bueno, Gardner, Prado, & Metzger, 2010).

We applied this framework to an amphibian metacommunity composed of 23 local communities encompassing a clear successional gradient classified in four major successional stages: young (2–5 years old) secondary forests, intermediate (13–28 years old) secondary forests, old‐growth forest fragments, and forest sites in a well‐preserved continuous forest, all located in the Lacandona rainforest, southeast Mexico. We studied amphibians due to their high dependence on environmental conditions, such as temperature, humidity, and presence of water bodies (Duellman & Trueb, 1994; Navas & Otani, 2007), and their low mobility compared with other vertebrates (Gibbons et al., 2000; Gibbs, 1998; Lemckert, 2004; Pittman, Osbourn, & Semlitsch, 2014; Popescu & Gibbs, 2010). Amphibians are also among the most threatened terrestrial vertebrates on earth (42% of the known the species are threatened; IUCN, 2017), mainly due to habitat loss and degradation (Vié, Craig, & Stuart, 2009). On the other hand, amphibians are poorly represented in natural protected areas (Nori et al., 2015), increasing the importance of secondary forests in their protection (Hernández‐Ordóñez, Urbina‐Cardona, & Martínez‐Ramos, 2015). We sampled amphibians between 2010 and 2012. We identified 15 ecologically relevant functional traits and used a time‐calibrated molecular phylogeny for the recorded species (Pyron & Wiens, 2011). With this information, we estimated 14 complementary metrics of taxonomic, functional, and phylogenetic diversity.

Because amphibians have narrow environmental tolerances and low dispersal capabilities, we expected a strong environmental signal (species sorting) for the three dimensions of diversity and weak or no evidence of mass effect. More specifically, we expected an increase in alpha diversity from secondary forests to old‐growth forests because the latter group presents more suitable conditions for coexistence of forest‐specialist species. A similar trend is expected for the functional richness (FR), evenness, divergence, and dispersion, as the availability and heterogeneity of forest habitats are greater in old‐growth stands, and therefore, more functions may co‐occur. Assuming that niche and ecological interactions can be conserved along the phylogeny (Gómez, Verdu, & Perfectti, 2010; Wiens et al., 2010), we expected that phylogenetic divergence increases and taxa relatedness decrease along the successional gradient, being higher in old‐growth forests (Nowakowski, Frishkoff, Thompson, Smith, & Todd, 2018). Yet, if species sorting shapes amphibian communities, we expected that each successional stage could support different subsets of the regional fauna, resulting in a very high *β‐*diversity at the regional scale.

## 2 METHODS

### 2.1 Study area

The study was conducted between July 2010 and June 2012 in the southeastern part of the Lacandona rainforest, Chiapas, Mexico (16°05′N and 16°57′N, 90°45′W and 91°30′W; Supporting Information Figure S1), which is located in the northwestern portion of the Mayan forest (Nations, Primack, & Bray, 1999). The original vegetation consists mainly of lowland tropical rainforests (Ibarra‐Manríquez & Martínez‐Ramos, 2002). Annual precipitation is approximately 3,000 mm, and annual temperature averages 25°C (van Breugel, Martínez‐Ramos, & Bongers, 2006). The dominant geomorphologic units are classified as topographically irregular areas at 115–300 m asl with small hills and valleys with sandy and limestone soils (INE, 2000). This region is composed of old‐growth forest fragments (>50% of land cover) and secondary forests, crops (maize, beans, rice and palm), cattle pastures, and oil palm plantations (Couturier, Núñez, & Kolb, 2012). In the north is the Montes Azules Biosphere Reserve, with an area of 310,000 ha (INE, 2000). This region maintains one of the most species‐rich communities of amphibians in the country (Hernández‐Ordóñez et al., 2015, 2014), with only a few species shared with the Petén region in Guatemala, and the Mayan mountains in Belize (Hernández‐Ordóñez et al., 2014).

### 2.2 Study design and environmental characterization

We selected 23 forest sites in lowland areas (160–214 m asl; Supporting Information Table S1): 13 secondary forests and six old‐growth forest fragments within the Marqués de Comillas region (a nonprotected region), and four sites within the Montes Azules Biosphere Reserve (Supporting Information Table S1; Supporting Information Figure S1). All secondary forests come from abandoned cornfields (van Breugel et al., 2006). For each site, we quantified 11 environmental and vegetation variables. During the amphibian survey, air temperature and relative humidity were measured at the center of each site with a HOBO Pro v.2 (Onset data logger), installed at 1.5 m above ground. To quantify vegetation characteristics between July and October 2010, we established twenty‐four 2 × 2‐m plots, separated at least 10 m from each other, and recorded the following variables: percentage of forest floor covered by leaf litter, the number of nonwoody plants (ferns, palms and aroids and heliconiines), percentage of ground area covered by graminoids and herbaceous creepers, and the number of seedlings and number of woody stems (>50 cm height) in three DBH (diameter at breast height in centimeters) categories: <3.0, 3.1 to 30, and >30 (Supporting Information Table S1). We used a PCA to reduce these environmental variables into a few composite variables (axes). We then used Spearman rank correlation to assess pairwise relationships between the main PCA axes and environmental variables (Supporting Information Table S2). The first two principal components explained 63.2% of total variation (Axis‐1, 48.3% and Axis‐2, 14.9%; Supporting Information Figure S2). Based on these results, we grouped the sites in four successional stages: six small (1.5–3 ha) and young (2–5 years of abandonment) secondary forests (YSF), seven intermediate (13–28 years old; 1–4 ha) secondary forests (ISF), six old‐growth forest fragments (FF) ranging from 10 to 70 ha, and four sites within the Montes Azules Biosphere Reserve (Supporting Information Figure S2). The average distance among sites was 1,756 m for YSF, 1,731 m for ISF, 4,570 m for FF, and 4,464 m for CF.

### 2.3 Field surveys

To increase sampling completeness, we performed two complementary sampling methods (Ribeiro‐Júnior, Gardner, & Ávila‐Pires, 2008). The first method was a visual survey (Crump & Scott, 1994). Each site was sampled seven times between 2011 and 2012, two times during the dry season (March–April 2011), two times during the rainy season (August‐September 2011), and three times at the end of the dry season and the beginning of rainy season (May–July 2012). Each sampling day was divided into two time periods: 10:00–13:30 hr (daytime) and 19:00–22:30 hr (night time). Thus, total sampling effort per site was 98 hr (7 hr × 7 days × two person). To ensure adequately spaced sampling, during the same field season, sites were sampled with intervals of 15 days between each other. Our sampling covered a maximum of 2 m above ground (Hernández‐Ordóñez et al., 2015; Urbina‐Cardona, Olivares‐Pérez, & Reynoso, 2006); therefore, our study focused on species that use the forest understory. This method is widely used in tropical community herpetofaunal studies (Crump & Scott, 1994; Doan, 2003) and is the most effective method to survey rainforest herpetofauna (Doan, 2003). As a second and complementary method, we set up two 30‐m drift fence traps (T, shape) for each site, separated by 50 m between each other. For each trap, we buried four 19 liter buckets at ground level, separated 10 m between each other (Corn, 1994). Drift fences were activated and were reviewed daily during the three field seasons, for a total of 115 days. Total sampling effort per site was 5,520 hr (115 days × 2 traps × 24 hr). This technique is suggested for recording many cryptic and fossorial species (Ribeiro‐Júnior et al., 2008). Species were identified based on field guides of the Mayan forest herpetofauna (Campbell, 1998; Lee, 1996,2000) and taxonomic names followed the nomenclature proposed by Frost (2016).

### 2.4 Species diversity

To ensure that species diversity was adequately assessed in each site, we calculated the sample coverage estimator for each forest site (Chao & Jost, 2012) using the iNext software (Chao, Ma, & Hsieh, 2016). This coverage estimator is sensitive to species with one (singletons) or two (doubletons) individuals (Chao & Jost, 2012). Sample coverage varied from 0.84 to 0.99 (mean ± *SE*, 0.95 ± 0.22) in YSF, 0.90 to 0.94 (0.93 ± 0.08) in ISF, 0.91 to 0.99 (0.95 ± 0.11) in FF, and 0.94 to 0.98 (0.97 ± 0.11) in CF (Supporting Information Table S3). Therefore, our sampling effort was adequate and our results were not biased by differences in sample completeness among sites (Chao & Jost, 2012).

For each site, we measured taxonomic diversity with Hill numbers (Chao, Chazdon, Colwell, & Shen, 2006; Tuomisto, 2010), which have the same unit (i.e., effective number of species) and are useful to assess patterns of species diversity giving different weights to species abundances. In particular, we considered Hill numbers of order 0 (*^0^Dα*, species richness), 1 (*^1^Dα*, exponential Shannon entropy), and 2 (*^2^Dα*, inverse Simpson concentration). *^0^Dα* is not sensitive to species abundance, giving a disproportionate weight to rare species. *^1^Dα* is interpreted as the number of common species in the communities. *^2^Dα* favors dominant species and is therefore interpreted as the number of very abundant or dominant species in the community (Chao et al., 2006). To establish differences in species composition between successional stages, we estimated the β‐diversity based on Hill numbers: *^q^D_β_* = *^q^D_γ_*/*^q^D_α,_* where *^q^D_γ_* is the accumulated (regional) number of species in all successional stages, and *^q^D_α_* is the mean number of species per successional stage. *^q^D_β_*is interpreted as the “effective number of completely distinct communities” (Jost, 2007), and it ranges between 1 (when all sites or communities are identical) and *N* (when all *N* sites are completely different from each other). These metrics were estimated using the entropart package for R to implement a function to construct a matrix containing β‐diversity values of each pairwise comparison within each successional stage (Marcon & Herault, 2015).

### 2.5 Functional diversity

We measured seven functional traits per species (body size, toe webbing, mouth width, leg length, dorsum skin thickness/type, respiration type, and fecundation type) and eight life‐history traits (male reproductive display for female response, male reproductive display site, fecundation site, egg laying site, parental care of clutches, daily activity, habitat during nonbreeding season, and number of habitats used in nonbreeding season; Supporting Information Table S4). These traits were morphological and physiological characteristics measured at individual levels without reference to the environment or any other level of organization, and they are related to individual growth, reproduction, and survival of species (Supporting Information Table S4) (Duellman & Trueb, 1994; Wells, 2007). Additionally, they explain amphibian functions within the ecosystem (Cortés Gómez, Ramírez Padilla, & Urbina Cardona, 2015; Cortes‐Gómez, Ruiz‐Agudelo, Valencia‐Aguilar, & Ladle, 2015), and some of these traits have been used to explain the impact of habitat perturbation on amphibian communities in tropical regions (Cruz‐Elizalde, Berriozabal‐Islas, Hernandez‐Salinas, Martinez‐Morales, & Ramirez‐Bautista, 2016; Díaz‐García, Pineda, López‐Barrera, & Moreno, 2017; Ernst, Linsenmair, & Rodel, 2006; Ribeiro, Colli, Batista, & Soares, 2017; Riemann, Ndriantsoa, Rödel, & Glos, 2017). To obtain body size, mouth width, and leg length, we measured specimens shelved at the National Collection of Amphibians and Reptiles, and conducted a review from published literature of species of the Mayan forest and other tropical regions in Mexico (Campbell, 1998; Duellman, 2001; Lee, 1996,2000), complementing this information with data from our field surveys (Supporting Information Table S4). To identify functional groups (FG) within Lacandona amphibian species, we constructed a functional dendrogram based on a species traits matrix (Supporting Information Table S5) using Euclidian distance and unweighted pair‐group arithmetic average clustering (Bihn, Gebauer, & Brandl, 2010). The data yielded a Cophenetic correlation coefficient of 0.87, which indicates that our dendrogram represents a realistic representation of natural variation (Petchey & Gaston, 2006).

To assess the statistical significance of the observed FG within amphibian species, we used a Euclidian distance matrix and a similarity test (ANOSIM; 999 permutations). We assessed the weight of functional traits within the formed groups, using a PCA, to reduced 15 traits into a few composite variables (axes). The first two principal components explained 54% of the total variation (Axis‐36% and Axis‐2, 18%; Supporting Information Figure S3). We estimated four functional diversity metrics, which are independent from each other, and provide complementary information about functional diversity within the community (Mouchet, Villeger, Mason, & Mouillot, 2010; Villeger, Ramos Miranda, Flores Hernandez, & Mouillot, 2010). We calculated FR, functional evenness (FE), functional divergence (FD) (Bihn et al., 2010; Villeger et al., 2010), and functional dispersion (FDis) (Laliberte & Legendre, 2010). FR represents the amount of functional space occupied by the species of the community in the space of functional traits; FE describes the evenness of abundance distribution in a functional trait space; FD is the degree to which the distribution of abundances maximizes the dispersion of the functional traits of a community and can be interpreted as a measure of functional similarity between dominant species (Villeger et al., 2010). Finally, FDis is the mean distance of each species to the centroid of all species in the multidimensional traits space (Laliberte & Legendre, 2010). All analyses were performed in R software, using the FD package in R (Casanoves, Pla, Rienzo, & Diaz, 2011; Laliberte & Legendre, 2010).

### 2.6 Phylogenetic diversity

To construct our regional phylogeny, we pruned a time‐calibrated global phylogeny containing 2,800 amphibian species (Pyron & Wiens, 2011), to include only the 30 species recorded during our surveys. To assess the phylogenetic structure of the 23 amphibian communities, we used Phylocom 4.2 software (Webb, Ackerly, & Kembel, 2008; Webb, Ackerly, McPeek, & Donoghue, 2002). For each community, we estimated the phylogenetic metrics proposed by Webb et al. (2008), using abundance data: mean pairwise distance (MPD), mean nearest taxon distance (MNTD), net relatedness index (NRI), and the nearest taxon index (NTI). The MPD is the mean phylogenetic distance observed in the community phylogeny and is based on the length on the phylogeny branches, between each pair of taxa in a sample. The MNTD is the mean phylogenetic distance to the nearest taxon within the community and is based on the length on the nearest taxon of each species within the phylogeny. The NTI is a standardized metric of the MPD of taxa in a sample and quantifies the overall clustering of taxa on a tree. The NRI is a standardized measure of the phylogenetic distance to the nearest taxon for each taxon in the sample and quantifies the extent of terminal clustering, independent of deep level clustering (Webb et al., 2002; Webb, Losos, & Agrawal, 2006). Positive values of NRI and NTI indicate phylogenetic clustering and negative values indicate overdispersion (Webb et al., 2002).

### 2.7 Relationships between species diversity and geographic and environmental data

We tested the isolated effect of intersite distance (spatial autocorrelation) and environmental variables (environmental autocorrelation) on community structure with Mantel tests (999 permutations and based on Pearson's correlation). The environmental matrix was based on the first two axes of the PCA (Supporting Information Figure S2). To assess the relative effect of environmental variables on species diversity while controlling for the effect of geographic distance, we also performed partial Mantel tests using the software Primer V7 (Clarke & Gorley, 2015).

### 2.8 Comparison between diversity and successional stages

To identify and test for any significant structure in the local communities, we used the similarity profile permutation test (SIMPROF), with 9,999 Monte Carlo simulations (Clarke, Somerfield, & Gorley, 2008). Similarity profile analysis examines whether observed similarities in data are smaller or larger than expected by chance. In this routine, the statistic Pi determines the deviation of the true profile from the mean permuted null distribution of the profile.

Furthermore, to explore species distribution across the ordination space, we used a principal coordinate analysis (PCoA), an unconstrained ordination of multivariate data, with the Bray–Curtis measure of species abundance data (Clarke et al., 2008). First, we transformed data to square root relative abundance values. The Bray–Curtis dissimilarity index was selected as the criterion of distance among sites (Anderson & Willis, 2003). This index is recommended for community data and has been used in previous herpetological studies (Urbina‐Cardona et al., 2006). In the PCoA, arrow vector orientation and length represents the association, direction, and strength between the species and the ordination axis (Anderson & Willis, 2003). The SIMPROF and PCoA were also carried out using the software Primer V7 (Clarke & Gorley, 2015). We used generalized linear models to test for differences in all diversity metrics between successional stages. Finally, we tested a second model, adding the waterbody type as a cofactor, to determine if there was an influence of waterbody type on our response variables. We fixed a Gaussian error distribution for all 14 metrics. All statistical analyses were performed using JMP® Version 13.0 (SAS Institute Inc., Cary, NC, 1989–2016).

## 3 RESULTS

**Figure 1:**
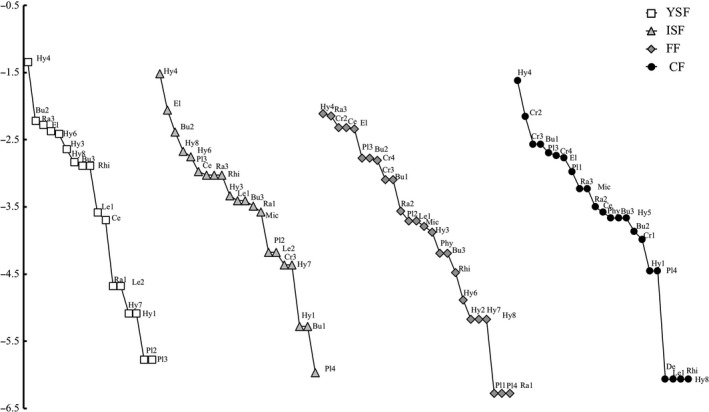
Species‐rank abundance curves for amphibian communities across four successional stages in southeastern Mexico. We separately show young (2–5 fallow age; YSF) and intermediate (9–28 fallow age; ISF) secondary forests, and old‐growth forest fragments (FF) and continuous forest (CF). The 10 species that occurred in all successional stages are shown with codes: Pl3, *Bolitoglossa rufescens;*Hy4, *Smilisca baudinii*; Hy8, *Tlalocohyla picta*; Bu2, *Incilius valliceps*; Bu3, *Rhinella horribilis*; Ra3, *Lithobates vaillanti*; Le1, *Leptodactylus fragilis*; El, *Eleutherodactylus leprus*; Ce, *Hyalinobatrachium fleischmanni*; Rhi, *Rhinophrynus dorsalis*

We recorded 1,672 individuals belonging to 30 amphibian species, 18 genera, 11 families, and three orders (Supporting Information Table S3). The most diverse family was Hylidae, with 8 species, and the most abundant species was *Smilisca baudinii* (Hylidae). Several families showed one single species (*Rhinophrynidae*, *Microhylidae*, *Centrolenidae*, *Dermophiidae*, *Eleutherodactylidae*, and *Phyllomedusidae*; Supporting Information Table S3). Ten species occurred along the entire gradient (Figure 1). There was only one singleton and no doubletons. Three species were exclusive to CF, one species was exclusive to FF, but none species occurred exclusively in YSF or ISF. We recorded no exotic species.

### 3.1 Species diversity

**Figure 2:**
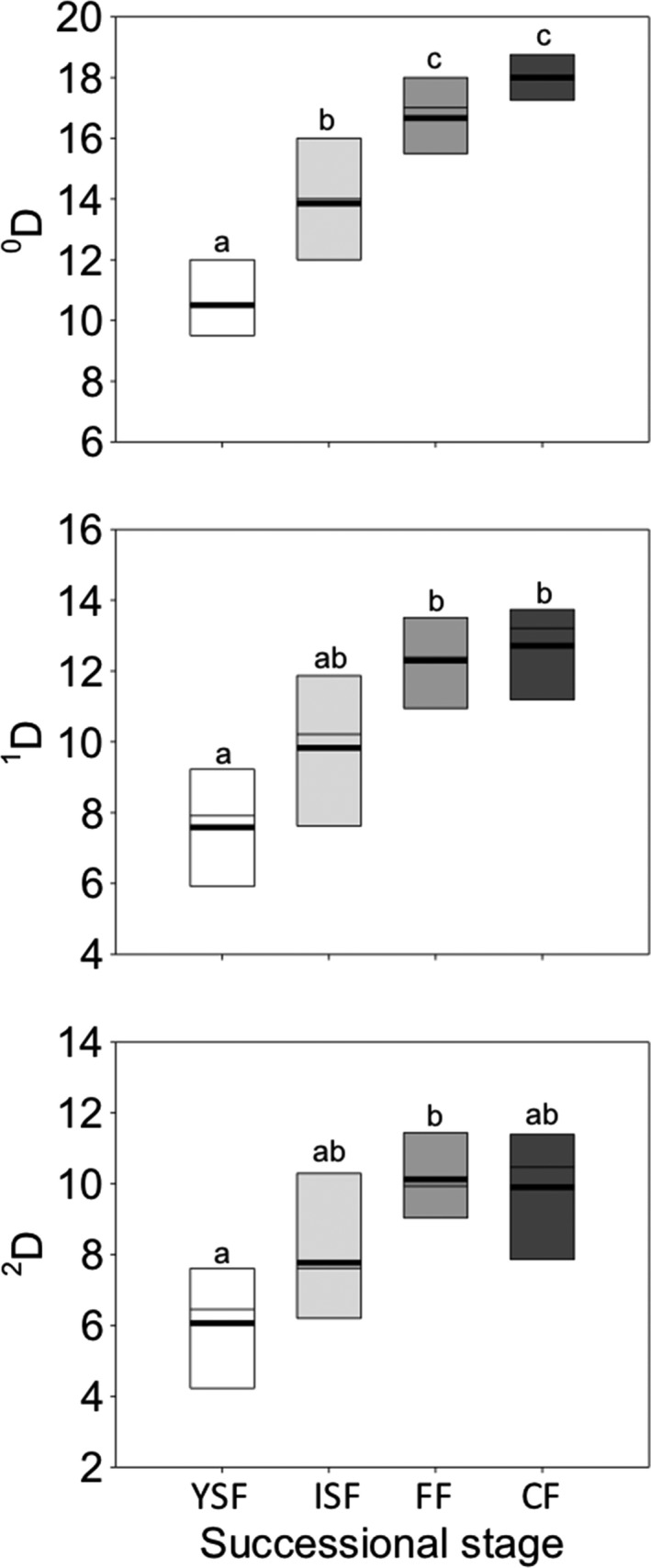
Box plots of alpha amphibian species diversity in young secondary forests (YSF, *n* = 6), intermediate secondary forests (ISF, *n* = 7), old‐growth forest fragments (FF, *n* = 6), and old‐growth continuous forests (CF, *n* = 4), southern Mexico. The mean (solid line), median (thin line), and 10th and 90th percentiles (boundaries of boxes) are indicated. Different letters indicate statistical differences between treatments. Metrics are described in detail in the Methods section

All study sites were mostly independent, as we only found a significant but weak spatial autocorrelation in *^2^Dα* (Table 1). However, 8 of 12 response variables were positively and significantly related to environmental variables (Table 1). All three metrics of species diversity (*^0^Dα*, *^1^Dα*, *^2^Dα*) increased from younger to older successional stages (Figure 2; Table 2). Species diversity in FF did not differ significantly from those in CF, but it was 54%–78% lower in YSF than in FF and CF (Figure 2). ISF showed intermediate values of species diversity, differing mainly in terms of species richness (Figure 2). Regarding β‐diversity among successional stages, we found values of 2.08, 1.80, and 1.67 communities for orders 0, 1, and 2, respectively. This indicates that β‐diversity was relatively low, as it can vary from 1 (when all communities are identical) to 4 (when all four communities are completely different from each other). In this sense, 10 species occurred in all successional stages (Supporting Information Table S3).

**Table 1:** Results of the Mantel tests (simple and partial) applied to species composition, species diversity, functional diversity, and phylogenetic diversity of amphibian communities in the Lacandona rainforest, Mexico

Diversity metric^a^	Geographic distance (Geo.dist)	Environmental distance (Env.dist)	Env.dist and Geo.dist
*^0^D_α_*	0.05^NS^	0.61**	0.61**
*^1^D_α_*	0.18^NS^	0.44**	0.45**
*^2^D_α_*	0.28*	0.33**	0.30*
FR	0.13^NS^	0.27*	0.13^NS^
FE	−0.13^NS^	0.008^NS^	−0.13^NS^
FD	0.16^NS^	−0.17^NS^	0.16^NS^
FDis	−0.10^NS^	0.63**	−0.14^NS^
MPD	−0.08^NS^	0.31**	−0.08^NS^
MNTD	−0.09^NS^	0.28**	−0.10^NS^
NTI	−0.02^NS^	0.12^NS^	−0.02^NS^
NRI	0.28^NS^	−0.01^NS^	0.28^NS^
Community structure	0.11^NS^	0.57**	0.58**

Notes

Values indicate *R*‐values (*Rho*). *N* = 23 local communities.

NS: not significant.

a Taxonomic diversity: species richness (*^0^D_α_*), exponential Shannon entropy (*^1^D_α_*), inverse Simpson concentration (*^2^D_α_*). Functional diversity: functional richness (FR), functional evenness (FE), functional divergence (FD), functional dispersion (FDis). Phylogenetic diversity: mean nearest taxon distance (MNTD), nearest taxon index (NTI), net relatedness index (NRI).

* *p* < 0.05.

** *p* < 0.01.

*** *p* < 0.001.

**Table 2:** Results of linear models applied to taxonomic species diversity (*^0^D_α_,^1^D_α_*and *^2^D_α_*), functional richness (FRric), evenness (FE), divergence (FD) and dispersion (Fdis), mean phylogenetic distance (MPD), mean nearest taxon distance (MNTD), net relatedness index (NTI) and nearest taxon index (NTI) of amphibian communities in the Lacandona rainforest, southern Mexico

Model	Successional stage	Waterbody
*^0^D_α_*	19.33***	1.18^ns^
*^1^D_α_*	7.65**	0.41^ns^
*^2^D_α_*	4.74*	0.12^ns^
FR	10.93**	2.1^ns^
FE	0.50^ns^	0.23^ns^
FD	1.82^ns^	1.01^ns^
FDis	15.02***	2.31^ns^
MPD	6.35**	0.35^NS^
MNTD	5.78**	4.31*
NRI	1.16^NS^	0.17^NS^
NTI	4.34*	2.84^NS^

Notes

Models include four levels of successional stage (young secondary forests, intermediate secondary forest, old‐growth forest fragment, and old‐growth continuous forest) and quality for three types of waterbody (pond, temporary, or permanent waterbody). Values indicate *F*‐values. *N* = 23 local communities.

ns: not significant.

* *p* < 0.05.

** *p* < 0.01.

*** *p* < 0.001.

**Figure 3:**
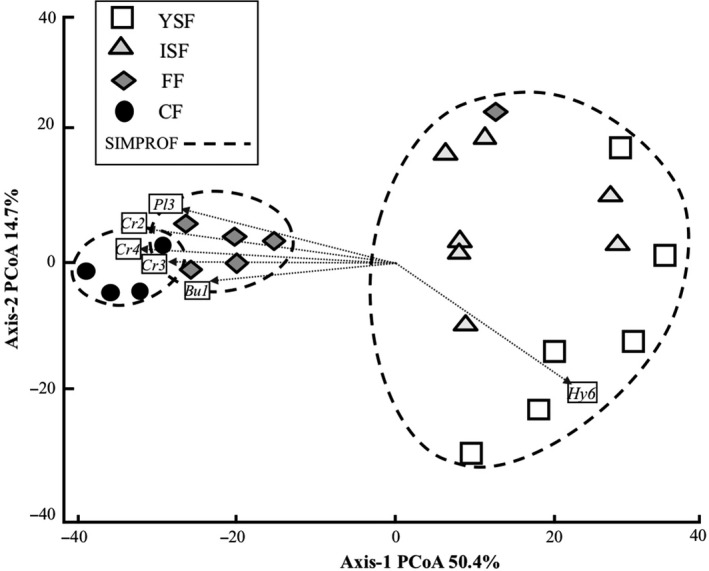
Principal coordinates analysis (PCoA) of amphibian communities along the successional gradient in the Lacandona rainforest, southern Mexico. Dashed lines indicate different groups in terms of community structure. Circles around the sites indicate the statistically significant groups and species codes indicate the associated species for the groups. White squares (YSF: young secondary forests); light gray triangles (ISF: intermediate secondary forests); dark gray rhombus (FF: old‐growth forest fragments); and black circles (CF: old‐growth continuous forest)

Community structure (species composition and numbers of individuals per specie) in the 23 sites was mostly independent of the spatial location, but significantly related to environmental characteristics (Table 1). The SIMPROF test and PCoA ordination revealed three broad groups (Figure 3). Axis‐1 explained 50.4% of the variance, and this analysis produced three clearly identified groups: secondary forests, forest fragments, and continuous forest (*Pi = *4.8, *p* = 0.001). Three craugastorid, one plethodontid, and one bufonid are associated with old‐growth forests, and one hylid is associated with secondary forests (Figure 3). Along axis‐2 (that explained 14.7% of the variance), young and intermediate secondary forests showed greater dispersion in community structure, even in the face of low β‐diversity. The type of the waterbodies (pond, temporary, or permanent waterbody) in the sites did not affect any metric of α‐diversity (Table 2).

### 3.2 Functional and phylogenetic diversity

The Euclidian distance dendrogram showed 11 FG, and the similarly test (ANOSIM) indicated significant differences among FG (*R*_statistic_ = 0.4; *p = *0.002). Most of the resulting FG were composed mainly of species in the same family or genus (Supporting Information Table S5; Supporting Information Figure S4), and in some cases, a single species was grouped alone (i.e., *Gymnopis syntrema*, *Smilisca cyanosticta*, *Hyalinobratachium fleischmani*). Traits, such as morphofunctional (i. e., body size, respiration type, mouth and length size, toe webbing and skin type) and reproductive (i. e., male reproductive display for female response, fecundation type), were the most important traits when grouping species (Supporting Information Figure S3). For example, respiration type, male reproductive display for female response, fecundation type, and male reproductive display site were the traits that determined FG3, which was composed of plethodontids, skin type, and egg laying site arranged FG6, composed by bufonids (Supporting Information Figures S3 and S4).

**Figure 4:**
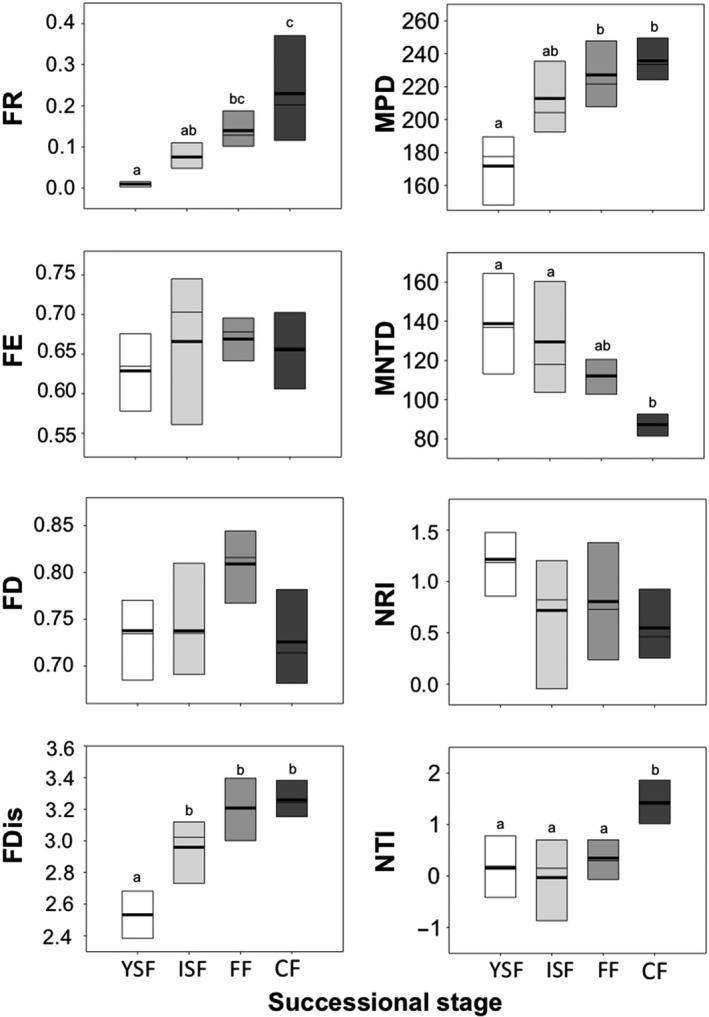
Functional and phylogenetic metrics of amphibian communities in young secondary forest (YSF, *n* = 6), intermediate secondary forests (ISF, *n* = 7), old‐growth forest fragments (FF, *n* = 6), and old‐growth continuous forests (CF, *n* = 4), Southern Mexico. The mean (solid line), median (thin line), and 10th and 90th percentiles (boundaries of boxes) are indicated. Different letters indicate statistical differences between treatments. Metrics are described in detail in the Methods section

For all functional and phylogenetic metrics, we did not find spatial autocorrelation among 23 sites (Table 1). FR increased significantly from YSF to old‐growth continuous forest (Figure 4; Table 2). FR values showed a lower variation in young secondary forest than in old‐growth continuous forest (Figure 4). FDis also increased in the same direction (Figure 4; Table 2), being particularly lower in young secondary forests. FE and FD did not respond significantly to the environmental gradient (Table 2).

Mean pairwise distance increased from the young secondary forest to the old‐growth continuous forest (Figure 4; Table 2), while the mean MNTD showed an opposite pattern (Figure 4). This result was expected as more lineages in the community decrease distances to nearest nonconspecifics. The differences in these metrics of phylogenetic divergence were higher when comparing the extremes of the gradient, although MNTD was lower in old‐growth continuous forests than in secondary forests.

In terms of phylogenetic structure, all local communities tended to be more related than random draws from the metacommunity, as indicated by positive values of the NTI. Nonetheless, this metric did not vary consistently along the successional gradient, suggesting that most lineages are well represented along the gradient (Table 2). On the other hand, the NTI remained similar across the young secondary forest, intermediate secondary forest, and old‐growth forest fragments (with positive and negative values), but it drastically increased in the old‐growth continuous forest, indicating higher relatedness closer to the tips of the phylogeny (i.e., within families and genera; Figure 4; Table 2). The type of the waterbodies in the forests only affected one the phylogenetic metric (MNTD; Table 2).

## 4 DISCUSSION

Our findings suggest that the diversity and structure of amphibian communities along a successional gradient in a fragmented tropical rainforest is largely explained by species sorting (i.e., environmental filtering). From young‐ to old‐growth forest patches, we observed (a) an increase in species diversity, regardless of species abundance; (b) a notable taxonomic segregation across successional stages; (c) an increase in FR and dispersion; (d) an increase in mean phylogenetic distance and nearest taxon index; and (e) a reduction in mean nearest taxon distance. While these taxonomic, functional, and phylogenetic results support a strong environmental signal in the structure of amphibian communities along environmental gradients (Hernández‐Ordóñez et al., 2015; Russildi, Arroyo‐Rodríguez, Hernandez‐Ordoñez, Pineda, & Reynoso, 2016; Suazo‐Ortuño et al., 2018), they also highlight the need for more integrated assessments of amphibian diversity that go beyond counting species and individuals. A clear phylogeny including over 2,800 frogs, salamanders, and caecilians of the world is available since 2011 (Pyron & Wiens, 2011), but to date, only four studies have evaluated the phylogenetic patterns of amphibian diversity along environmental gradients (Barratt et al., 2017; Martins et al., 2016; Nowakowski et al., 2018; Ribeiro et al., 2017). Similarly, ecological, physiological, and life‐history trait data are increasingly available (Oliveira, Sao‐Pedro, Santos‐Barrera, Penone, & Costa, 2017; Ribeiro et al., 2017), but their use in amphibian community ecology still relies on a few traditional, nonintegrative approaches.

It is not surprising that amphibian diversity is organized into different environmental conditions along the successional gradient. Although waterbodies (i.e., covariate) did not affect the diversity metrics (except the MNTD), it is well known that amphibian demography is strongly linked to environmental conditions (Navas & Otani, 2007; Owen, 1989). For example, large and thick‐skinned species such as the Cane Toad (*Rhinella horribilis*), southern Gulf Coast Toad (*Incilius valliceps*), and the Veined Tree Frog (*Trachycephalus typhonius*; FG6; Supporting Information Table S5) are associated with open and disturbed areas. While small, thin‐skinned and direct‐developing species such as craugastorids (FG9) and plethodontids (FG3; Supporting Information Table S5) are associated with closed canopies and humid sites (Campbell, 1998; Lee, 2000), as their desiccation and overheating tolerance increase with higher surface area‐to‐volume ratios (Pfeifer et al., 2017). Also, both families (FG) are strongly affiliated with old‐growth forests (Nowakowski et al., 2018). This narrow environmental tolerance causes the amphibian metacommunity in regenerating forests to be largely structured by species sorting. Other groups with less environmental restrictions and increased dispersal abilities (i.e., bufonids‐FG6; leptodactylids‐FG7; some hylids‐FG11) are more likely to be influenced by mass effects (Gonzalez, Lawton, Gilbert, Blackburn, & Evans‐Freke, 1998).

Although most of our findings indicate that species are sorted across their spatial niches, we cannot disregard the fact that mass effects are involved in the metacommunity structure. The main reason for that was the relatively low β‐diversity at the regional scale. In fact, 10 out of 30 species (33.3%) occurred in all successional stages. Together, these findings suggest that immigration and emigration events are common across forests successional stages, allowing a third of the species to occur elsewhere in the study area. The taxonomic segregation revealed by the ordination appears to contradict the low β‐diversity, but it reflects changes in the species relative abundance along the successional gradient, which again support the species sorting perspective.

Both the mass effect and species sorting perspectives assume that there are intrinsic differences among local sites in terms of their attributes such that different species might be favored at different sites (Leibold et al., 2004). Both perspectives also assume that there are trade‐offs in the abilities of species to perform well under different habitat conditions (Leibold et al., 2004). The main differences rely on the role of dispersal (i.e., movement of individuals from one site to another) in connecting patches of differing quality, which is more relevant in the mass effect perspective. The perspectives also rely on the time scales between local population dynamics and colonization‐extinction dynamics, which are assumed to be separated in the species sorting perspective and simultaneous in the mass effect perspective (Leibold et al., 2004). We hypothesize that the amphibians of our study area are substantially influenced by patch quality and slightly influenced by dispersal events. Apparently, species are sorted along the gradient according to their niches, but earlier successional stages are supplemented by immigrants from older successional stages (Marsh & Trenham, 2001), allowing both local colonization and extinction along the successional gradient. For example, the higher individual number and *^0^Dα* of salamanders (FG3; Figure 1) in old‐growth forests (FF and CF) than in secondary forests could be explained by the functional traits of this FG that shares respiration type, egg laying site, leg length. In this sense, those traits could be impeding the establishment of these species in the secondary forests. In addition, three of four craugastorid species (FG9) are exclusive to old‐growth forests (Figure 3), species in this group, such as salamanders lay their eggs under leaf litter (Supporting Information Table S5). Therefore, the high number of individuals of these FG in the old‐growth forests are determinants of the species structure in these successional stages (Figure 3). On the other hand, the *^0^Dα* of hylids is higher in the secondary forests (Supporting Information Table S3); in this sense, FG11 contains five hylid species (Supporting Information Table S5). This FG is defined by traits such as leg and mouth length and reproductive mode strategies (Supporting Information Table S5) but that does not make them dependent on high rates of relative humidity, only to the presence of lentic waterbodies, such as ponds (Campbell, 1998; Duellman, 2001; Lee, 2000).

The varied responses of diversity metrics to forest succession emphasize the need for estimating sets of metrics rather than a few taxonomic metrics (Andrade et al., 2015; Pausas & Verdu, 2010). Looking exclusively at the local (*α*) component of diversity indicates that secondary forests are much poorer in diversity than old‐growth forests. However, in agreement with Sfair, Arroyo‐Rodriguez, Santos and Tabarelli (2016), when we take the between‐community (*β*) component into account, secondary forests show different species (biotic differentiation), thus contributing to increase species diversity at the regional scale (Sfair et al., 2016). In this sense, secondary forests can have a high conservation value, with the potential to provide habitat for a large portion of the taxonomic, functional, and phylogenetic amphibian diversity observed in the old‐growth continuous forest (Dent & Wright, 2009). Nonetheless, these forests are usually slashed and burned at intervals smaller than 10 years (van Breugel et al., 2006); thus, long‐term amphibian conservation cannot take advantage of secondary forests until this fallow period is considerably extended, to decades if possible.

The diversity metrics of forest fragments were almost entirely similar to those of continuous forest, indicating a great tolerance of amphibian communities to forest isolation. This result is consistent with previous assessments of tree species (Hernández‐Ruedas et al., 2014) and reptile species (Russildi et al., 2016) the region, emphasizing the need for conserving as many forest fragments as possible in the region. In this sense, several species are listed in the IUCN (*Eleutherodactylus leprus*, *Bolitoglossa mulleri*, and *Craugastor alfredi* are listed in the IUCN red list as vulnerable; *Incilius campbelli*, *S. cyanosticta*, and *Craugastor laticeps* are listed as near threatened; and *Craugastor palenque* and *G. syntrema*are listed as data deficient; IUCN, 2017), and the population trends for 34.3% of the species are decreasing (Hernández‐Ordóñez et al., 2014), with most of these species are exclusively in old‐growth forests (Supporting Information Table S3). Preventing deforestation is therefore mandatory in the region.

## CONFLICT OF INTEREST

None declared.

## AUTHORS' CONTRIBUTIONS

VHR, OHO, BAS, VAR, and MMR conceived the ideas and designed methodology; OHO collected the data; RAP and BAS constructed the species phylogeny cladogram; BAS, NUC, and OHO analyzed the data; BAS, OHO, VHR, GPO, and VAR led the writing of the manuscript. All authors contributed critically to the drafts and gave final approval for publication.

## Data Availability

Raw data (Supporting Information Tables S1 and S3) will be available in Dryad Digital Repository https://doi.org/10.5061/dryad.2v8rq57.
