# Supplementary material for: Species sorting and mass effect along forest succession: Evidence from taxonomic, functional, and phylogenetic diversity of amphibian communities
Source: Ecol Evol. 2019 Apr 8;9(9):5206–18. doi: 10.1002/ece3.5110 (PMC6509387; doi:10.1002/ece3.5110)
Supplement: Supplementary file 1 [file ECE3-9-5206-s001.docx]

Supporting Information for:

**Species sorting and mass effect along forest succession: evidence from taxonomic, functional, and phylogenetic diversity of amphibian communities**

Omar Hernández-Ordóñez, Bráulio A. Santos, R. Alexander Pyron, Víctor Arroyo-Rodríguez, José N. Urbina-Cardona, Gabriela Parra-Olea, Miguel Martínez-Ramos, Víctor H. Reynoso

**This file includes:**

Table S1. Environmental characteristics and measured variables of each site.

Table S2. Spearman rank correlation between PCA axes site environmental characteristics.

Table S3. Amphibians number of individuals per specie and the eleven metrics calculated for the three dimensions, per site.

Table S4. Traits used to calculate functional diversity metrics.

Table S5. Species functional trait matrix

Figure S1. Location of the study area, southeast Mexico.

Figure S2. PCA ordination plot of environmental variables in relation with sites.

Figure S3. PCA ordination plot of amphibian species and functional traits

Figure S4. Dendrogram of the functional groups of amphibians

References

**TABLE S1** Site characteristics in the Lacandona rainforest: fallow age (years) for secondary forests; area (ha); altitude (m asl); topography, valley= v or small hill= h. Waterbody type, different water body types were categorized: pond (po); permanent stream (ps) and temporal stream (ts). Hum= Humidity; Temp= Temperature (**ºC)**; Herb= percentage of ground area covered by graminoids and herbaceous creepers; Leaf-litter= percentage of forest floor covered by litter-fall; Palm= number of palms; Fern= number of ferns; Aroid/helicon= number of aroids and helicons; Seedling= number of seedlings; WP1 = number of woody stems > 50 cm height but < 3.0 cm DBH; WP2 = number of woody stems 3.1 – 30 cm DBH, WP3 = number of woody stems > 30 cm DBH. YSF: young secondary forests; ISF: intermediate secondary forests; FF: old growth forests fragments; CF: continuous old growth forest.

| **Site** | YSF1 | YSF2 | YSF3 | YSF4 | YSF5 | YSF6 | ISF1 | ISF2 | ISF3 | ISF4 | ISF5 | ISF6 | ISF7 | FF1 | FF2 | FF3 | FF4 | FF5 | FF6 | CF1 | CF2 | CF3 | CF4 |
| --- | --- | --- | --- | --- | --- | --- | --- | --- | --- | --- | --- | --- | --- | --- | --- | --- | --- | --- | --- | --- | --- | --- | --- |
| **Fallow age** | 3 | 4 | 5 | 3 | 2 | 5 | 13 | 15 | 21 | 28 | 15 | 19 | 14 | >50 | >50 | >50 | >50 | >50 | >50 | >50 | >50 | >50 | >50 |
| **Area** | 2 | 3 | 1.5 | 1 | 1.5 | 1.5 | 4 | 1 | 4 | 5.5 | 5.8 | 2.3 | 5 | 10.3 | 10.8 | 70.0 | 19.5 | 18.7 | 48.1 | >300,000 | >300,000 | >300,000 | >300,000 |
| **Altitude** | 181 | 171 | 153 | 183 | 160 | 172 | 175 | 170 | 175 | 181 | 206 | 160 | 184 | 186 | 189 | 169 | 192 | 172 | 203 | 176 | 180 | 174 | 214 |
| **Topography** | v | v | v | h | h | v | h | v | v | v | h | v | v | v | v | v | v | h | h | h | v | h | v |
| **Waterbody** | p | ts | p | ps | ps | ps | ps | ts | ps | ts | ts | p | p | ps | p | ps | ps | ps | ps | ts | ps | ps | ps |
| **Temp** | 28.3 | 28.8 | 28.0 | 25.9 | 25.2 | 25.7 | 25.2 | 24.9 | 25.4 | 25.1 | 25.3 | 25.3 | 25.2 | 24.7 | 25.2 | 24.8 | 25.0 | 25.0 | 23.7 | 24.6 | 24.4 | 24.3 | 23.8 |
| **Hum** | 76.9 | 75.0 | 79.8 | 83.8 | 83.3 | 83.8 | 86.3 | 85.8 | 85.0 | 88.1 | 88.5 | 86.2 | 87.5 | 91.5 | 88.1 | 92.0 | 88.8 | 88.8 | 94.9 | 92.1 | 93.1 | 92.1 | 93.2 |
| **Herb** | 46.0 | 70.3 | 68.3 | 42.2 | 29.0 | 39.6 | 32.6 | 36.3 | 24.6 | 28.0 | 22.2 | 20.0 | 27.8 | 22.8 | 28.8 | 24.3 | 5.1 | 30.6 | 6.7 | 35.9 | 35.9 | 15.1 | 9.9 |
| **Leaf-litter** | 41.0 | 15.4 | 18.1 | 18.4 | 17.6 | 47.7 | 43.7 | 37.0 | 53.4 | 42.1 | 41.2 | 55.9 | 44.8 | 60.3 | 50.9 | 48.0 | 55.3 | 49.9 | 30.3 | 36.7 | 40.8 | 49.6 | 30.8 |
| **Palm** | 0.0 | 0.0 | 0.0 | 0.4 | 0.2 | 0.0 | 0.4 | 0.2 | 0.1 | 0.6 | 0.3 | 0.4 | 0.5 | 2.7 | 0.3 | 1.1 | 0.3 | 1.2 | 3.2 | 4.3 | 5.2 | 4.6 | 1.7 |
| **Fern** | 0.3 | 0.9 | 0.5 | 1.6 | 0.8 | 1.1 | 0.3 | 0.3 | 1.1 | 0.0 | 0.1 | 1.5 | 0.0 | 1.9 | 1.3 | 2.5 | 3.3 | 4.5 | 1.4 | 2.1 | 2.9 | 3.5 | 2.2 |
| **Seedling** | 9.3 | 3.6 | 3.1 | 2.5 | 3.4 | 5.2 | 11.2 | 3.8 | 11.0 | 16.7 | 13.4 | 5.0 | 4.0 | 13.3 | 12.8 | 11.4 | 17.8 | 10.3 | 13.5 | 8.9 | 8.7 | 15.9 | 14.3 |
| **WP1** | 4.1 | 3.8 | 4.0 | 1.7 | 1.6 | 2.1 | 5.5 | 1.9 | 3.1 | 5.1 | 3.8 | 3.4 | 2.5 | 3.3 | 3.8 | 3.5 | 3.7 | 4.8 | 5.2 | 2.4 | 5.0 | 4.3 | 4.6 |
| **WP2** | 1.4 | 1.0 | 0.3 | 0.9 | 2.0 | 1.0 | 1.8 | 2.1 | 1.8 | 2.1 | 1.4 | 2.4 | 1.5 | 1.8 | 1.8 | 1.2 | 1.4 | 1.7 | 1.6 | 0.8 | 2.0 | 1.2 | 1.6 |
| **WP3** | 0.0 | 0.1 | 0.0 | 0.1 | 0.2 | 0.1 | 0.3 | 0.1 | 0.1 | 0.4 | 0.4 | 0.2 | 0.2 | 0.8 | 0.4 | 0.8 | 0.6 | 0.6 | 0.7 | 0.8 | 0.8 | 0.9 | 0.8 |
| **Aroid/helicon** | 3.0 | 4.7 | 5.3 | 2.4 | 2.0 | 2.6 | 2.5 | 3.0 | 4.9 | 2.0 | 2.9 | 7.5 | 5.5 | 1.4 | 4.7 | 4.5 | 0.8 | 4.3 | 2.9 | 4.3 | 1.5 | 1.4 | 1.3 |

**TABLE S2** Spearman rank correlation values resulting from pairwise comparisons between: PCA score axes and site environmental characteristics. Hum= Humidity; Temp= Temperature (**ºC)**; Herb= percentage of ground area covered by graminoids and herbaceous creepers; Leaf-litter= percentage of forest floor covered by litter-fall; Palm= number of palms; Fern= number of ferns; Aroid/helicon= number of aroids and helicons; Seedling= number of seedlings; WP1 = number of woody stems > 50 cm height but < 3.0 cm DBH; WP2 = number of woody stems 3.1 – 30 cm DBH, WP3 = number of woody stems > 30 cm DBH.

| **Environmental variable** | **PCA axis-1** | **PCA axis-2** | **PCA axis-3** |
| --- | --- | --- | --- |
| Hum | -0.85*** | -0.21 | 0.34 |
| Temp | 0.92*** | 0.003 | -0.24 |
| Herb | -0.82 | -0.38* | 0.07 |
| Leaf-litter | 0.55 | 0.53 | 0.04 |
| Palm | 0.69** | -0.49 | -0.21 |
| Fern | 0.62 | -0.37 | -0.19 |
| Aroid/helicon | -0.36 | 0.41 | -0.33 |
| Seedling | 0.78 | 0.04 | 0.5 |
| WP1 | 0.43* | -0.12 | 0.74 |
| WP2 | 0.35 | 0.70* | 0.12 |
| WP3 | 0.91** | -0.32 | -0.06 |

Asterisks (*) indicate the level of significance: * = p < 0.05, ** = p < 0.01, *** = p < 0.001

**TABLE S3** Amphibians number of individulas per specie, in parentheses species codes for PCoA, dendrogram and PCA (functional groups). Coverage sample and taxonomic, functional and phylogenetic metrics calculated per each site. YSF: young secondary forests; ISF: intermediate secondary forests; FF: old growth forests fragments; CF: continuous old growth forest.

| **Site** | YSF1 | YSF2 | YSF3 | YSF4 | YSF5 | YSF6 | ISF1 | ISF2 | ISF3 | ISF4 | ISF5 | ISF6 | ISF7 | FF1 | FF2 | FF3 | FF4 | FF5 | FF6 | CF1 | CF2 | CF3 | CF4 |
| --- | --- | --- | --- | --- | --- | --- | --- | --- | --- | --- | --- | --- | --- | --- | --- | --- | --- | --- | --- | --- | --- | --- | --- |
| Order Gymnophiona |  |  |  |  |  |  |  |  |  |  |  |  |  |  |  |  |  |  |  |  |  |  |  |
| Family Dermophiidae |  |  |  |  |  |  |  |  |  |  |  |  |  |  |  |  |  |  |  |  |  |  |  |
| *Gymnopis syntrema* (De) | 0 | 0 | 0 | 0 | 0 | 0 | 0 | 0 | 0 | 0 | 0 | 0 | 0 | 0 | 0 | 0 | 0 | 0 | 0 | 1 | 0 | 0 | 0 |
| Order Urodela |  |  |  |  |  |  |  |  |  |  |  |  |  |  |  |  |  |  |  |  |  |  |  |
| Family Plethodontidae |  |  |  |  |  |  |  |  |  |  |  |  |  |  |  |  |  |  |  |  |  |  |  |
| *Bolitoglossa mexicana* (Pl1) | 0 | 0 | 0 | 0 | 0 | 0 | 0 | 0 | 0 | 0 | 0 | 0 | 0 | 0 | 0 | 0 | 0 | 0 | 1 | 2 | 10 | 8 | 2 |
| *Bolitoglossa mulleri* (Pl2) | 0 | 1 | 0 | 0 | 0 | 0 | 0 | 2 | 0 | 2 | 1 | 1 | 0 | 2 | 1 | 1 | 3 | 4 | 2 | 0 | 0 | 0 | 0 |
| *Bolitoglossa rufescens* (Pl3) | 0 | 1 | 0 | 0 | 0 | 0 | 3 | 1 | 2 | 5 | 4 | 2 | 3 | 3 | 2 | 2 | 5 | 13 | 8 | 7 | 6 | 9 | 7 |
| *Oedipina elongata* (Pl4) | 0 | 0 | 0 | 0 | 0 | 0 | 0 | 0 | 1 | 0 | 0 | 0 | 0 | 0 | 0 | 0 | 0 | 1 | 0 | 2 | 1 | 1 | 1 |
| Order Anura |  |  |  |  |  |  |  |  |  |  |  |  |  |  |  |  |  |  |  |  |  |  |  |
| Family Phyllomedusidae |  |  |  |  |  |  |  |  |  |  |  |  |  |  |  |  |  |  |  |  |  |  |  |
| *Agalychnis callidryas* (Phy) | 0 | 0 | 0 | 0 | 0 | 0 | 0 | 0 | 0 | 0 | 0 | 0 | 0 | 0 | 0 | 8 | 0 | 0 | 0 | 3 | 8 | 0 | 0 |
| Family Hylidae |  |  |  |  |  |  |  |  |  |  |  |  |  |  |  |  |  |  |  |  |  |  |  |
| *Dendropsophus microcephalus* (Hy1) | 1 | 1 | 0 | 0 | 0 | 0 | 0 | 0 | 0 | 0 | 0 | 1 | 1 | *0* | 0 | 0 | 0 | 0 | 0 | 0 | 5 | 0 | 0 |
| *Dendropsophus ebraccatus* (Hy2) | 0 | 0 | 0 | 0 | 0 | 0 | 0 | 0 | 0 | 0 | 0 | 0 | 0 | 0 | 0 | 0 | 0 | 0 | 3 | 0 | 0 | 0 | 0 |
| *Scinax staurfferi* (Hy3) |  |  |  |  |  |  |  |  |  |  |  |  |  |  |  |  |  |  |  |  |  |  |  |
| *Smilisca baudinii* (Hy4) | 7 | 0 | 2 | 4 | 3 | 7 | 0 | 4 | 4 | 1 | 2 | 2 | 1 | 2 | 2 | 1 | 1 | 3 | 2 | 0 | 0 | 0 | 0 |
| *Smilisca cyanosticta* (Hy5) | 5 | 6 | 24 | 25 | 17 | 8 | 6 | 12 | 20 | 10 | 11 | 23 | 4 | 13 | 6 | 4 | 25 | 11 | 5 | 23 | 22 | 18 | 22 |
| *Tachycephalus thyphonius* (Hy6) | 4 | 2 | 6 | 4 | 11 | 3 | 1 | 5 | 6 | 2 | 0 | 8 | 3 | 0 | 0 | 0 | 2 | 1 | 1 | 0 | 0 | 0 | 0 |
| *Tlalocohyla loquax* (Hy7) | 2 | 1 | 0 | 0 | 0 | 0 | 0 | 0 | 1 | 4 | 0 | 0 | 0 | 1 | 2 | 0 | 0 | 0 | 0 | 0 | 0 | 0 | 0 |
| *Tlalocohyla picta* (Hy8) | 12 | 0 | 0 | 2 | 4 | 1 | 3 | 1 | 16 | 5 | 0 | 1 | 1 | 0 | 1 | 2 | 0 | 0 | 0 | 0 | 0 | 0 | 1 |
| Family Craugastoridae |  |  |  |  |  |  |  |  |  |  |  |  |  |  |  |  |  |  |  |  |  |  |  |
| *Craugastor alfredi* (Cr1) | 0 | 0 | 0 | 0 | 0 | 0 | 0 | 0 | 0 | 0 | 0 | 0 | 0 | 0 | 0 | 0 | 0 | 0 | 0 | 1 | 3 | 3 | 1 |
| *Craugastor laticeps* (Cr2) | 0 | 0 | 0 | 0 | 0 | 0 | 0 | 0 | 0 | 0 | 0 | 0 | 0 | 4 | 0 | 2 | 13 | 14 | 19 | 15 | 7 | 18 | 10 |
| *Craugastor loki* (Cr3) | 0 | 0 | 0 | 0 | 0 | 0 | 0 | 2 | 0 | 2 | 1 | 0 | 0 | 3 | 4 | 3 | 7 | 4 | 3 | 6 | 12 | 12 | 3 |
| *Craugastor palenque* (Cr4) | 0 | 0 | 0 | 0 | 0 | 0 | 0 | 0 | 0 | 0 | 0 | 0 | 0 | 1 | 0 | 5 | 8 | 11 | 7 | 9 | 10 | 5 | 4 |
| Family Eleutherodactylidae |  |  |  |  |  |  |  |  |  |  |  |  |  |  |  |  |  |  |  |  |  |  |  |
| *Eleutherodactylus leprus* (El) | 8 | 2 | 2 | 6 | 1 | 11 | 13 | 10 | 6 | 4 | 11 | 2 | 4 | 14 | 7 | 8 | 10 | 7 | 5 | 6 | 3 | 11 | 7 |
| Family Rhinophrynidae |  |  |  |  |  |  |  |  |  |  |  |  |  |  |  |  |  |  |  |  |  |  |  |
| *Rhinophrynus dorsalis* (Rhi) | 2 | 0 | 8 | 0 | 5 | 3 | 0 | 0 | 1 | 3 | 0 | 13 | 2 | 0 | 2 | 1 | 0 | 1 | 2 | 0 | 0 | 1 | 0 |
| Family Microhylidae |  |  |  |  |  |  |  |  |  |  |  |  |  |  |  |  |  |  |  |  |  |  |  |
| *Gastrophryne elegans* (Mic) | 0 | 0 | 0 | 0 | 0 | 0 | 1 | 3 | 2 | 3 | 1 | 0 | 1 | 2 | 3 | 1 | 3 | 2 | 1 | 9 | 0 | 2 | 6 |
| Family Centrolenidae |  |  |  |  |  |  |  |  |  |  |  |  |  |  |  |  |  |  |  |  |  |  |  |
| *Hyalinobatrachium fleischmanni* (Ce) | 0 | 0 | 0 | 3 | 5 | 0 | 13 | 2 | 4 | 0 | 0 | 0 | 0 | 12 | 0 | 3 | 17 | 6 | 14 | 3 | 3 | 4 | 2 |
| Family Bufonidae |  |  |  |  |  |  |  |  |  |  |  |  |  |  |  |  |  |  |  |  |  |  |  |
| *Incilius campbelli* (Bu1) | 0 | 0 | 0 | 0 | 0 | 0 | 0 | 0 | 0 | 1 | 1 | 0 | 0 | 1 | 0 | 5 | 12 | 3 | 3 | 7 | 17 | 8 | 1 |
| *Incilius valliceps* (Bu2) | 5 | 9 | 10 | 1 | 8 | 2 | 7 | 2 | 4 | 2 | 7 | 7 | 7 | 2 | 6 | 3 | 14 | 4 | 4 | 2 | 3 | 1 | 3 |
| *Rhinella horribilis* (Bu3) | 3 | 4 | 1 | 4 | 3 | 3 | 5 | 1 | 1 | 1 | 2 | 0 | 3 | 0 | 1 | 6 | 0 | 1 | 0 | 1 | 5 | 3 | 2 |
| Family Leptodactylidae |  |  |  |  |  |  |  |  |  |  |  |  |  |  |  |  |  |  |  |  |  |  |  |
| *Leptodactylus fragilis* (Le1) | 2 | 1 | 1 | 0 | 2 | 3 | 1 | 1 | 2 | 2 | 3 | 2 | 2 | 1 | 3 | 6 | 1 | 2 | 0 | 0 | 1 | 0 | 0 |
| *Leptodactylus melanonotus* (Le2) | 0 | 0 | 0 | 1 | 2 | 0 | 0 | 0 | 0 | 0 | 2 | 1 | 3 | 0 | 0 | 0 | 0 | 0 | 0 | 0 | 0 | 0 | 0 |
| Family Ranidae |  |  |  |  |  |  |  |  |  |  |  |  |  |  |  |  |  |  |  |  |  |  |  |
| *Lithobates brownorum* (Ra1) | 1 | 3 | 0 | 0 | 0 | 0 | 0 | 1 | 3 | 3 | 0 | 3 | 2 | 0 | 1 | 0 | 0 | 0 | 0 | 0 | 0 | 0 | 0 |
| *Lithobates maculatus* (Ra2) | 0 | 0 | 0 | 0 | 0 | 0 | 0 | 0 | 0 | 0 | 0 | 0 | 0 | 2 | 0 | 0 | 9 | 0 | 4 | 2 | 0 | 10 | 1 |
| *Lithobates vaillanti* (Ra3*)* | 0 | 0 | 0 | 11 | 10 | 12 | 1 | 5 | 13 | 0 | 0 | 0 | 0 | 17 | 0 | 7 | 22 | 12 | 4 | 4 | 4 | 7 | 2 |
| **n** | 52 | 31 | 54 | 61 | 71 | 53 | 54 | 52 | 86 | 50 | 46 | 66 | 37 | 80 | 41 | 68 | 152 | 100 | 88 | 105 | 126 | 124 | 75 |
| **S.obs** | 12 | 11 | 8 | 10 | 12 | 10 | 11 | 15 | 16 | 16 | 12 | 13 | 14 | 16 | 14 | 18 | 16 | 18 | 18 | 19 | 18 | 18 | 17 |
| **C.hat** | 0.96 | 0.84 | 0.96 | 0.97 | 0.99 | 0.98 | 0.93 | 0.91 | 0.95 | 0.94 | 0.92 | 0.94 | 0.90 | 0.95 | 0.91 | 0.94 | 0.99 | 0.96 | 0.97 | 0.97 | 0.98 | 0.98 | 0.94 |
| **^0^D** | 12.0 | 11.0 | 8.0 | 10.0 | 12.0 | 10.0 | 11.0 | 15.0 | 16.0 | 16.0 | 12.0 | 13.0 | 14.0 | 16.0 | 14.0 | 18.0 | 16.0 | 18.0 | 18.0 | 19.0 | 18.0 | 18.0 | 17.0 |
| **^1^D** | 9.4 | 8.0 | 4.9 | 6.3 | 9.2 | 7.9 | 7.6 | 10.5 | 10.2 | 13.1 | 8.2 | 7.4 | 11.9 | 9.9 | 11.3 | 14.7 | 12.0 | 13.1 | 12.8 | 12.9 | 13.8 | 13.5 | 10.6 |
| **^2^D** | 7.8 | 6.2 | 3.7 | 4.4 | 7.6 | 6.7 | 6.2 | 8.0 | 7.6 | 10.8 | 6.4 | 5.2 | 10.3 | 7.5 | 9.6 | 12.9 | 10.3 | 10.9 | 9.6 | 9.6 | 11.3 | 11.4 | 7.3 |
| **FR** | 0.0 | 0.0 | 0.0 | 0.0 | 0.0 | 0.0 | 0.0 | 0.1 | 0.1 | 0.1 | 0.0 | 0.1 | 0.1 | 0.1 | 0.1 | 0.2 | 0.1 | 0.2 | 0.1 | 0.4 | 0.2 | 0.2 | 0.1 |
| **FE** | 0.6 | 0.6 | 0.5 | 0.6 | 0.7 | 0.7 | 0.6 | 0.7 | 0.6 | 0.8 | 0.5 | 0.7 | 0.7 | 0.7 | 0.7 | 0.7 | 0.6 | 0.7 | 0.7 | 0.6 | 0.7 | 0.6 | 0.7 |
| **FD** | 0.7 | 0.7 | 0.7 | 0.7 | 0.7 | 0.8 | 0.8 | 0.7 | 0.7 | 0.7 | 0.7 | 0.7 | 0.8 | 0.8 | 0.8 | 0.8 | 0.8 | 0.8 | 0.9 | 0.7 | 0.7 | 0.8 | 0.7 |
| **FDis** | 2.4 | 2.6 | 2.3 | 2.4 | 2.7 | 2.8 | 3.2 | 3.0 | 2.6 | 3.1 | 3.0 | 2.7 | 3.1 | 3.3 | 3.0 | 3.1 | 3.0 | 3.5 | 3.4 | 3.2 | 3.3 | 3.4 | 3.1 |
| **MPD** | 142.2 | 184.4 | 170.5 | 149.9 | 187.1 | 196.5 | 168.9 | 204.3 | 192.4 | 263.1 | 198.7 | 235.4 | 227.1 | 219.8 | 223.4 | 197.7 | 211.3 | 270.3 | 240.3 | 231.8 | 221.5 | 254.4 | 235.1 |
| **MNTD** | 112.3 | 113.4 | 161.9 | 129.1 | 144.5 | 171.3 | 142.9 | 114.0 | 101.0 | 117.9 | 103.7 | 166.5 | 160.3 | 106.9 | 130.6 | 117.2 | 97.2 | 116.3 | 104.5 | 88.3 | 79.7 | 86.8 | 94.0 |
| **NTI** | 0.8 | 0.8 | 0.0 | 0.4 | -0.3 | -0.7 | -0.1 | 0.4 | 0.7 | 0.2 | 0.9 | -0.9 | -1.5 | 0.6 | -0.1 | -0.1 | 1.1 | 0.0 | 0.6 | 1.3 | 2.0 | 1.5 | 0.9 |
| **NRI** | 2.1 | 1.1 | 0.6 | 1.3 | 1.2 | 0.9 | 1.3 | 1.0 | 1.2 | 0.0 | 0.8 | 0.0 | 0.8 | 0.6 | 0.8 | 2.1 | 1.1 | -0.3 | 0.4 | 0.6 | 1.0 | 0.2 | 0.3 |

**TABLE S4** Traits used to calculate functional diversity metrics; methods to calculated, relevance with the performance of amphibian species and relation with ecological process.

| **Trait** | **Attributes** | **Definition** | **Method** | **Relevance for the species** | **Ecological process** |
| --- | --- | --- | --- | --- | --- |
| Body size (SVL) | Small (20-40mm); Medium (41-61mm); Large (61-80mm); Extra-large, EL(>81mm) | Snout vent length (mm) | We measured snout to its vent (mm) in individuals shelved in the National Collection of Amphibians and Reptiles (UNAMN) from Lacandona region and nearby zones | Large species are associated with high water deficit areas, whereas small species are usually found in humid areas. Size reflect effects of reduced surface to volume ratios in larger species to control both heat and water balance (Olalla-Tarraga *et al.* 2009) | Related to nutrient cycling and energy flow through of trophic webs as predator or prey. Larger species contribute more biomass to the ecosystem than small species and consume a greater amount of prey |
| Toe webbing on posterior feet | Abs=Absent | Webbing is absent on feet | We classified Lacandona species based on Cortés Gómez, Ramírez Padilla and Urbina Cardona (2015), in case of caecilids, we assigned without feet attribute | Toe webbing indicates the habitat that species inhabits or used during the breeding season. Extended webbing is present in species with aquatic habitats, while moderated webbing species are mainly arboreal species. Basal and absent webbing are from terrestrial species. This trait is related with thermoregulation and hydroregulation protection against predation (Cortés Gómez, Ramírez Padilla & Urbina Cardona 2015) | Related to nutrient cycling and energy flow through of trophic webs as predator or prey. Also, is related with the water cycle. |
|  | Bas=Basal | Webbing below the middle of the 4th finger |  |  |  |
|  | Mod=Moderate | Webbing is up to the middle of the 4th finger |  |  |  |
|  | Ext= Extended | Webb absent on posterior legs |  |  |  |
|  | Nf=Not feet | Organisms without legs (caecilians) |  |  |  |
| Mouth width in proportion to SVL | S (Small)= < 20% of the SVL | Proportion of the mouth width is related with the SVL | We measured specimens shelved in National Collection of Amphibians and Reptiles (UNAM) from Lacandona region and nearby zones. Values used for this study was means of adult males and females. Also, we calculated the proportion of mouth width in relation with the SVL | Is related in the foraging strategies of amphibian species. Organisms with small mouth is related with species that consumed smaller preys (specialist) and large mouth species could have consumed a diversity of preys (Toft 1981) | Related to nutrient cycling and energy flow through of trophic webs as predator |
|  | M (Medium)= 21-30% of the SVL |  |  |  |  |
|  | L (Large)= >31% SVL |  |  |  |  |
| Dorsum skin thickness and texture | 1= thin/smooth | skin is thin, and texture is smooth | We classified Lacandona species based on Cortés Gómez, Ramírez Padilla and Urbina Cardona (2015) and review the description of species and field guides (Campbell 1998; Lee 2000) | Thickness and texture of skin is related with the resistance to desiccation, where species with thick and granulated or postulated skin are more resistant to desiccation than thin and smooth species water loss rates is higher (Toledo & Jared 1993). This trait is related with thermoregulation and hydroregulation protection against predation (Cortés Gómez, Ramírez Padilla & Urbina Cardona 2015) | Related to nutrient cycling and energy flow through of trophic webs as predator or prey. Also, is related with the water cycle |
|  | 2= thin/granular | skin is thin, and texture is granular |  |  |  |
|  | 3= thin/granular-tuberculate | skin is thin, and texture is granular with some scattered tubercles |  |  |  |
|  | 4= thin/smooth-granular | skin is thin, and texture is granular with some scattered granules |  |  |  |
|  | 5= thin/postulated | skin is thin and composed of pustules |  |  |  |
|  | 6= thick/ smooth-tuberculate | skin is thick, and texture is smooth with some scattered tubercles |  |  |  |
|  | 7= thick/ tuberculate | skin is thick, and texture is tuberculate |  |  |  |
|  | 8=thick/ postulated | skin is thick and composed of pustules |  |  |  |
|  | 9= thin/tuberculate | skin is thin, and texture is tuberculate |  |  |  |
|  | 10= thin/ scale | skin is thin, and texture of dorsum is composed by scales |  |  |  |
| Leg length | NL=not legs | Proportion of the mouth width is related with the SVL | We measured posterior leg lengths (sum of tibia, femur and tarsus) specimens from the National Collection of Amphibians and Reptiles (UNAMN) from Lacandona region and nearby zones. Values used for this study are average values of adult males and females. Also, we calculated the proportion of mouth width in relation with the SVL | Posterior legs length, are related with the dispersion capacity and the strata that organism exploit (Pittman *et al.* 2008);where species with large legs can move longer distances or cover other strata such as undergrowth and canopy (i.e. Hylids) | Related to nutrient cycling and energy flow through of trophic webs as prey |
|  | 1= < 30% of the SLV |  |  |  |  |
|  | 2= 31-50 of the SVL |  |  |  |  |
|  | 3 =>51% SVL |  |  |  |  |
| Respiration type | 1= Predominally pulmonary | Species with lungs | We classified Lacandona species based on information about families and genera of amphibian species (Wells 2007) | Plethodontids skin breathe predominantly through their skin s related with low oxygen levels (i.e. leaflitter) also these organisms requires high humidity and low temperatures microhabitats (Wells 2007). Anurans and caecilids could inhabits in a width range harsh conditions | Amphibian respiration is related with the oxygen cycle within the ecosystem |
|  |  |  |  |  |  |
|  | 2= Predominally cutaneous | Species that breaths through skin |  |  |  |
| Fertilization | Int= internal | Is when during fertilization occurs cloacal opposition | We classified Lacandona species-based information about orders and family fertilization type (Beck 1998; Wells 2007) | Fertilization type is related with the balance among energy used for reproduction and the number of offspring | Traits related to nutrient cycling and energy flow through of trophic webs as prey |
|  |  |  |  |  |  |
|  | Ext= external | This fertilization type y more related with anuran species and is not present cloacal opposition |  |  |  |
| Male reproductive display for female response | Ac= Acoustic | Male strategy to attract females during breeding season is by acoustic callings | We classified Lacandona species based and review of ecological data of families, genera and species (Donnelly & Guyer 1994; Duellman & Trueb 1994; Campbell 1998; Lee 2000; Wells 2007; Vitt & Caldwell 2014)  Categories were established based on Duellman and Trueb (1994) and (Cortés Gómez, Ramírez Padilla & Urbina Cardona 2015) | The advertisement strategy implies behavioral, physiological and ecological differences (Wells 2007). This trait is related to the differential energy balance to produce offspring (Ribeiro (Ribeiro *et al.* 2017) |  |
|  | Horm= Hormonal | Male strategy to attract females during breeding season is by |  |  |  |
| Male reproductive display and fertilization site | 1= Understory above water bodies. | Male advertisement or copulation occurs on understory strata above water bodies (streams or ponds) |  | Display is related with the microhabitat and strata that exploit during the breeding season (Wells 2007). In some male reproductive display site is different with fertilization site that implies an energetic cost and depredation risk (Duellman & Trueb 1994) |  |
|  | 2= In to the water. | Male advertisement or copulation occurs in to water bodies (streams or ponds), usually individuals are floating |  |  |  |
|  | 3= Terrestrial. | Male advertisement or copulation occurs on terrestrial strata (rocks, leaf-litter, logs) |  |  |  |
|  | 4= Terrestrial next to waterbody. | Male advertisement or copulation occurs on terrestrial strata (rocks, leaf-litter, logs), but near streams (0-15 meters) |  |  |  |
|  | 5= Fossorial | Male advertisement or copulation occurs underground and leaf-litter |  |  |  |
|  | 6= Arboreal/Understory. | Male advertisement or copulation occurs between understory and arboreal strata |  |  |  |
|  | 7= Understory and terrestrial. | Male advertisement or copulation occurs between terrestrial and understory |  |  |  |
| Laying site | 1= eggs deposited out of the water with direct development. | eggs are deposited of the water mainly into leaf-litter or rocks. Offspring born fully developed |  | In some species laying site is different with fertilization site that implies an energetic cost; also, some species present some strategies to prevent the eggs desiccation (Duellman & Trueb 1994) |  |
|  | 2= eggs deposited in open water (streams and ponds), tadpoles develop in open water. | Eggs are deposited in open water (streams and ponds), and tadpoles develop in open water |  |  |  |
|  | 3=eggs deposited in open water or in tree cavities, tadpoles develop therein | Eggs are deposited in open water or in tree cavities, tadpoles develop therein |  |  |  |
|  | 4= Eggs deposited on vegetation, tadpoles develop in open water | Eggs deposited on vegetation, and after born tadpoles fall in to the waterbodies and develop therein |  |  |  |
|  | 5= eggs deposited in foam nest on or near water, tadpoles develop in water | Adults make up a foam nest were laid their eggs |  |  |  |
|  | 6= viviparity | Retention and growth of the fertilized egg within the maternal body |  |  |  |
| Clutches parental care | Pres= Present | Some form of parental care present, such as egg attendance |  | Parental care increases the probability that the clutches cannot be preyed |  |
|  |  |  |  |  |  |
|  | Abs= Absent | No form of parental care. Clutches and tadpoles are left unattended |  |  |  |
|  |  |  |  |  |  |
| Daily activity | Noc=nocturnal | Adults more active during the night | We classified Lacandona species based and review of ecological data of families, genera and species (Donnelly & Guyer 1994; Duellman & Trueb 1994; Campbell 1998; Lee 2000; Zug, Vitt & Caldwell 2001; Wells 2007; Vitt & Caldwell 2014) Also, we included data of field surveys in Lacandona region. We separated the habitat use of breeding season of the non-breeding season; due most species in the region use water bodies for their reproduction but some genera and families does not | Daily activity is the day period where organisms are more active. Also, this could be related with the kinds of prey and predator with which the organism interacts | This trait is related with the contribution in biomass and energy balance across the time |
|  |  |  |  |  |  |
|  |  |  |  |  |  |
|  |  |  |  |  |  |
|  |  |  |  |  |  |
|  |  |  |  |  |  |
|  |  |  |  |  |  |
|  |  |  |  |  |  |
|  |  |  |  |  |  |
|  | Cat=cathemeral | Adults more active during the night and sometimes at day (morning or crepuscular) |  |  |  |
| Habitat during not-breeding season | Arb= Arboreal | Arboreal species |  | Most species could be recorder in more than two vertical strata, during not breeding season, but other species do not used waterbodies for breeding | Traits related to nutrient cycling and energy flow through of trophic webs as prey |
|  | Fos= fossorial | Species adapted to digging and life underground |  |  |  |
|  | Und= understory | Understory species. |  |  |  |
|  | Ter= terrestrial | Terrestrial species |  |  |  |
|  | TerUnd= terrestrial/understory | Species that used daily terrestrial and understory strata |  |  |  |
|  | UndTer= understory/arboreal | Species that used daily understory and arboreal strata |  |  |  |
|  | TerWb= terrestrial next water bodies | Terrestrial species closed related with streams |  |  |  |
| Number of used habitats in not breeding season | Number of habitats where species have been recorded | Despite species preferring one habitat, some species could be recorder in more than two vertical strata |  |  |  |

**TABLE S5** Functional trait matrix, into parenthesis is the species code for dendrogram and PCA of the functional group figures (Figure S3, S4). Functional groups are the suggested by dendrogram and verified by ANOSIM.

|  | **Functional group** | **Body size (Bs)^a^** | **Toe webbing (Tw)^b^** | **Mouth width (Mw)^c^** | **Leg length (Ll)^d^** | **Dorsum skin thickness/type (St)^e^** | **Respiration type (Rt)^f^** | **Fertilization type (Ft)^g^** | **Male reproductive display for female response (Mdr)^h^** | **Male reproductive display site (Mds)^i^** | **Fertilization site (Fs)^j^** | **Egg laying site (Ls)^k^** | **Parental care clutches (Pc)^l^** | **Daily activity (Da)^m^** | **Habitat during non-breeding season (Hnb)^n^** | **Number habitats used in non-breeding season (Hn)^o^** |
| --- | --- | --- | --- | --- | --- | --- | --- | --- | --- | --- | --- | --- | --- | --- | --- | --- |
| *Gymnopis syntrema* (De) | FG1 | EL | NF | S | NL | 10 | Lung | Int | Hor | Fos | Fos | Viv | Abs | Cath | Fos | 2 |
| *Smilisca cyanosticta* (Hy5) | FG2 | L | Mod | L | EL | 1 | Lung | Ext | Ac | ArbUnd | ArbUnd | TreeWbTad | Abs | Noc | UndArb | 1 |
| *Bolitoglossa mexicana* (Pl1) | FG3 | M | Ext | M | S | 1 | Skin | Int | Hor | TerUnd | TerUnd | TerDirDev | Abs | Noc | TerUnd | 3 |
| *Bolitoglossa mulleri* (Pl2) | FG3 | M | Ext | M | M | 1 | Skin | Int | Hor | TerUnd | TerUnd | TerDirDev | Abs | Noc | TerUnd | 3 |
| *Bolitoglossa rufescens* (Pl3) | FG3 | S | Ext | M | S | 1 | Skin | Int | Hor | TerUnd | TerUnd | TerDirDev | Abs | Noc | TerUnd | 3 |
| *Oedipina elongata* (Pl4) | FG3 | M | Ext | S | S | 1 | Skin | Int | Hor | TerUnd | TerUnd | TerDirDev | Abs | Noc | TerUnd | 1 |
| *Craugastor alfredi* (Cr1) | FG4 | S | Bas | L | EL | 3 | Lung | Ext | Ac | TerUnd | Ter | TerDirDev | Pres | Noc | UndArb | 3 |
| *Eleutherodactylus leprus* (El) | FG4 | S | Abs | L | EL | 3 | Lung | Ext | Ac | TerUnd | Ter | TerDirDev | Pres | Noc | TerUnd | 3 |
| *Lithobates maculatus* (Ra2) | FG5 | L | Ext | L | L | 9 | Lung | Ext | Ac | InWb | InWb | TerWbTad | Abs | Noc | TerWb | 1 |
| *Lithobates vaillanti* (Ra1) | FG5 | L | Ext | L | EL | 9 | Lung | Ext | Ac | InWb | InWb | TerWbTad | Abs | Noc | TerWb | 1 |
| *Rhinophrynus dorsalis* (Rhi) | FG5 | M | Ext | M | L | 9 | Lung | Ext | Ac | InWb | InWb | TerWbTad | Abs | Noc | Fos | 2 |
| *Trachycephalus typhonius* (Hy6) | FG6 | L | Mod | L | L | 5 | Lung | Ext | Ac | InWb | InWb | TerWbTad | Abs | Noc | UndArb | 2 |
| *Incilius campbelli* (Bu1) | FG6 | L | Bas | L | L | 6 | Lung | Ext | Ac | TerNxWb | InWb | TerWbTad | Abs | Cath | TerWb | 2 |
| *Incilius valliceps* (Bu2) | FG6 | M | Bas | L | L | 7 | Lung | Ext | Ac | TerNxWb | InWb | TerWbTad | Abs | Cath | Ter | 2 |
| *Rhinella horribilis* (Bu3) | FG6 | L | Bas | L | L | 8 | Lung | Ext | Ac | TerNxWb | InWb | TerWbTad | Abs | Cath | Ter | 1 |
| *Leptodactylus fragilis* (Le1) | FG7 | S | Bas | L | L | 3 | Lung | Ext | Ac | TerNxWb | TerNxWb | FoamWbTad | Pres | Noc | Ter | 2 |
| *Leptodactylus melanonotus* (Le2) | FG7 | S | Bas | L | L | 3 | Lung | Ext | Ac | TerNxWb | TerNxWb | FoamWbTad | Pres | Cath | Ter | 3 |
| *Agalychnis callidryas* (Phy) | FG8 | M | Mod | L | EL | 1 | Lung | Ext | Ac | UndWb | UndWb | UndWbTad | Abs | Noc | Arb | 1 |
| *Hyalinobatrachium fleischmanni*  (Ce) | FG8 | S | Ext | L | EL | 1 | Lung | Ext | Ac | UndWb | UndWb | UndWbTad | Pres | Cath | Arb | 2 |
| *Craugastor laticeps* (Cr2) | FG9 | S | Bas | L | EL | 2 | Lung | Ext | Ac | Ter | Ter | TerDirDev | Pres | Cath | Ter | 2 |
| *Craugastor loki* (Cr3) | FG9 | S | Abs | L | EL | 4 | Lung | Ext | Ac | Ter | Ter | TerDirDev | Pres | Cath | Ter | 1 |
| *Craugastor palenque* (Cr4) | FG9 | M | Bas | L | EL | 4 | Lung | Ext | Ac | TerNxWb | TerNxWb | TerDirDev | Pres | Noc | TerWb | 1 |
| *Gastrophyne elegans* (Mic) | FG10 | S | Bas | M | L | 1 | Lung | Ext | Ac | InWb | InWb | TerWbTad | Abs | Noc | Ter | 3 |
| *Dendropsophus ebraccatus*  (Hy2) | FG10 | S | Mod | L | L | 1 | Lung | Ext | Ac | UndWb | UndWb | UndWbTad | Abs | Noc | UndArb | 3 |
| *Dendropsophus microcephalus*  (Hy1) | FG11 | S | Mod | L | EL | 1 | Lung | Ext | Ac | UndWb | InWb | TerWbTad | Pres | Noc | UndArb | 1 |
| *Scinax staufferi* (Hy3) | FG11 | S | Mod | L | L | 1 | Lung | Ext | Ac | UndWb | InWb | TerWbTad | Abs | Noc | UndArb | 1 |
| *Tlalocohyla loquax* (Hy7) | FG11 | S | Ext | L | L | 4 | Lung | Ext | Ac | UndWb | InWb | TerWbTad | Abs | Noc | UndArb | 1 |
| *Tlalocohyla picta* (Hy8) | FG11 | S | Mod | L | EL | 1 | Lung | Ext | Ac | UndWb | InWb | TerWbTad | Abs | Noc | UndArb | 2 |
| *Lithobates brownorum* (Ra1) | FG11 | L | Ext | L | EL | 1 | Lung | Ext | Ac | InWb | InWb | TerWbTad | Abs | Noc | TerWb | 1 |
| *Smilisca baudinii*  (Hy4) | FG11 | M | Mod | L | L | 1 | Lung | Ext | Ac | UndWb | InWb | TerWbTad | Abs | Cath | UndArb | 1 |

^a^ Body Size: S, Small (20-40mm); M, Medium (41-61mm); L, Large (61-80mm); Large (L)= 61-80mm; Extra-large, EL(>81mm)

^b^ Toe Webbing: Abs, Absent; Bas, Basal; Mod, Moderate; Ext, Extended; NF, not feet

^c^ Mouth width: S, small (<20%); M, medium (21-30%); L, Large (>30%)

^d^ Leg length in proportion with SVL: NL, Not legs; S, Small (<1-35% of the SLV); M, Medium (36-80%); L, large (81-130%); EL, Extra-Large (>130%)

^e^ Dorsum skin thickness/type: 1= thin/smooth; 2= thin/granular; 3= thin/granular-tuberculated; 4= thin/smooth-granular; 5= thick/postulated; 6= thick/ smooth to tuberculated; 7= thick/ tuberculated; 8= thick/ pustulated; 9= thin/tuberculated; 10= thin/ scale.

^f^ Respiration type: Lung, predominantly pulmonary; Skin, predominantly cutaneous.

^g^ Fertilization type: Int, internal; Ext: external

^h^ Male reproductive display for female response: Ac, acoustic; Hor, hormonal.

^i^ Male reproductive display site and ^j^ fertilization site: UndWb, Understory above waterbody; InWb; Into waterbody; Ter, Terrestrial; TerNxWb, Terrestrial next to waterbody, Fos, Forsorial; ArbUnd, Arboreal or Understory; TerUnd, Terrestrial or Understory

^k^ Laying site: TerDirDev, Terrestrial with direct development; Viv, viviparous; TreeWbTad, Tree waterbody tadpoles; TerWbTad, Terrestrial waterbody tadpoles; UndWbTad, Understory waterbody tadpoles; FoamWbTad, Foam nets waterbody tadpoles

^l^ Clutches parental care: Pres= Present; Abs= Absent.

^m^ Daily activity: Noc= nocturnal; Cath=cathemeral.

^n^ Basic habitat in not breeding season: Arb= arboreal; Fos= fossorial; Und= understory; Ter= terrestrial; TerUnd= terrestrial/understory; Und/arb= understory/arboreal; TerWb= terrestrial next water bodies.


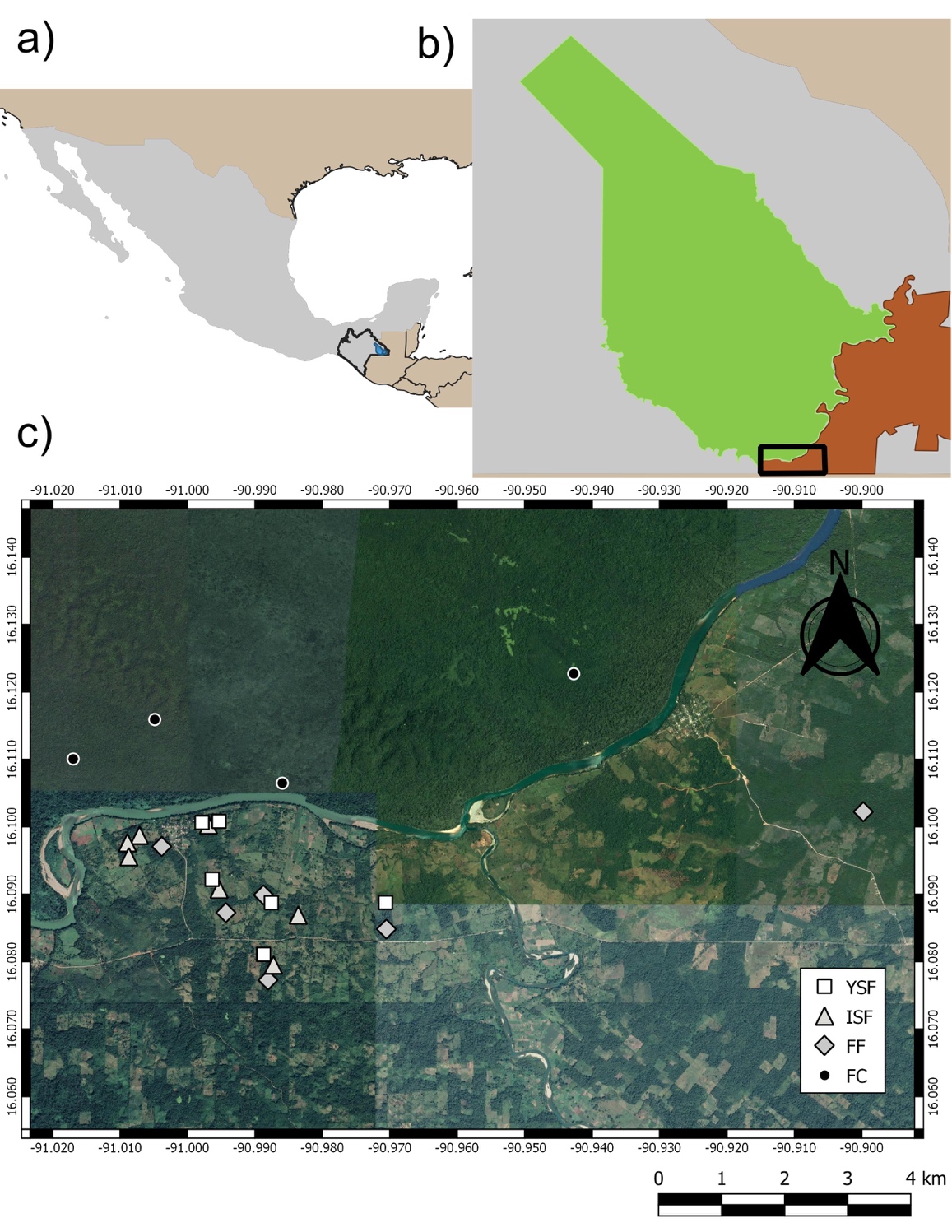


**Figure S1** Location of the study area, southeast Mexico; a) location of the Lacandona region (blue polygon), in Chiapas state, Mexico; b) location of study area within the Lacandona region; and c) study sites

**
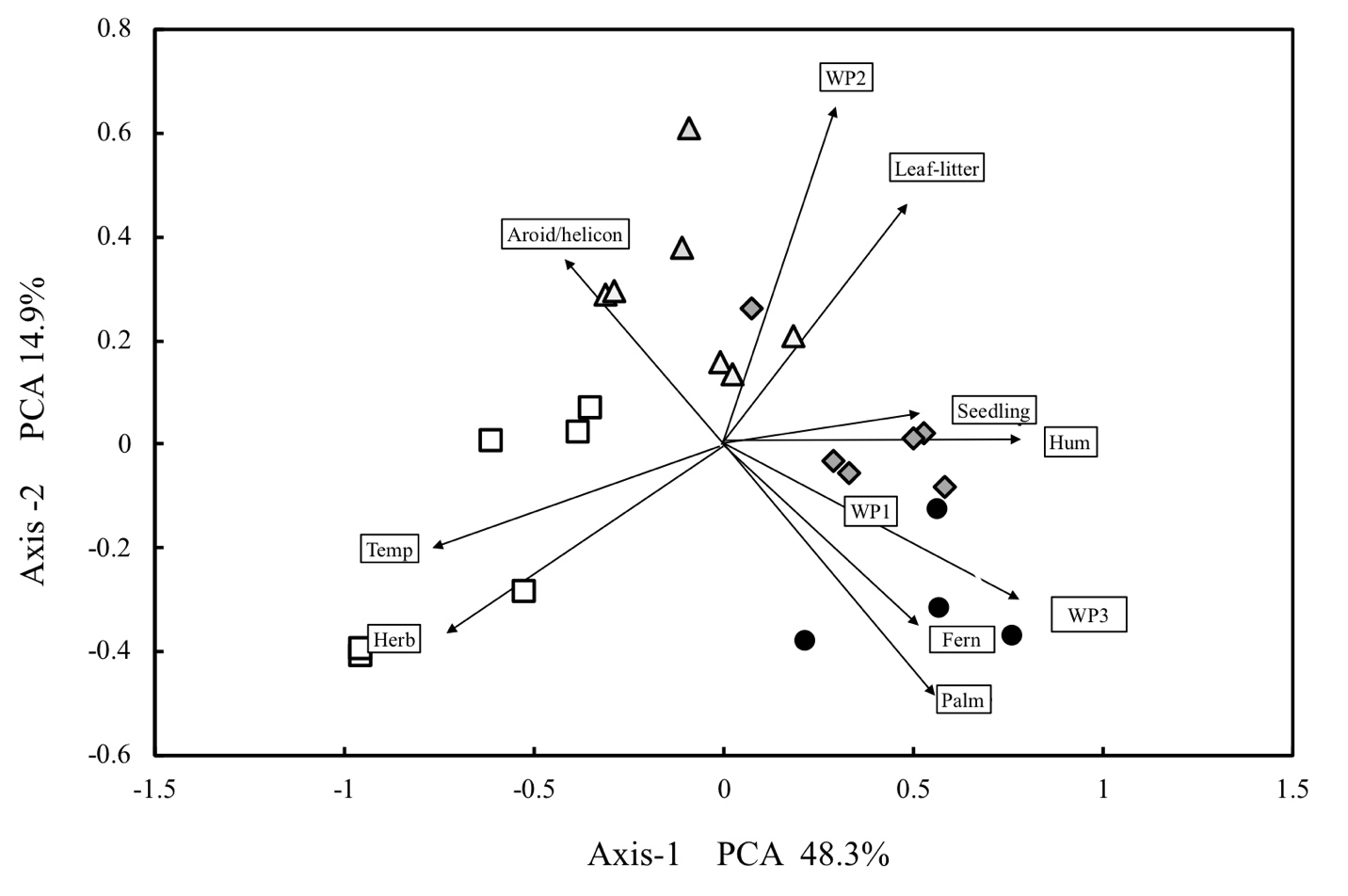
**

**Figure S2** PCA ordination representing the 11 environmental variables in relation to sites within four different successional categories. Inside the boxes are the environmental variables and the *r* value and its statistical significance is showed in Table S2. White squares (YSF: young secondary forests); light gray triangles (ISF: intermediate secondary forests); dark gray rhombus (FF: old growth forests fragments); and black circles (CF: continuous old growth forest)


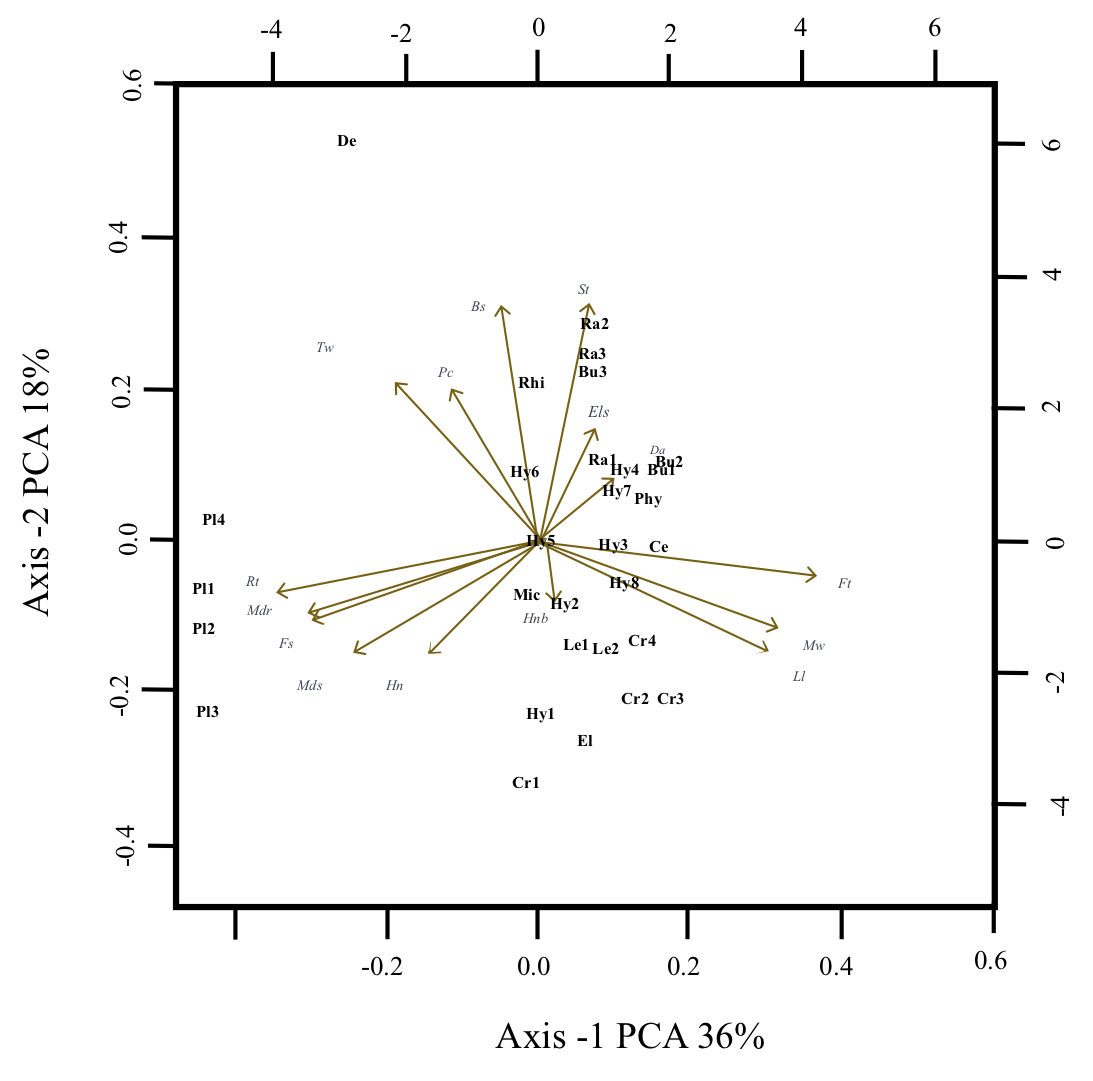


**FIGURE S3** Principal Components Analysis of Lacandona amphibian species and functional traits; bold letters indicate species (codes are indicated in Table S4) and italic letters indicated trait

**
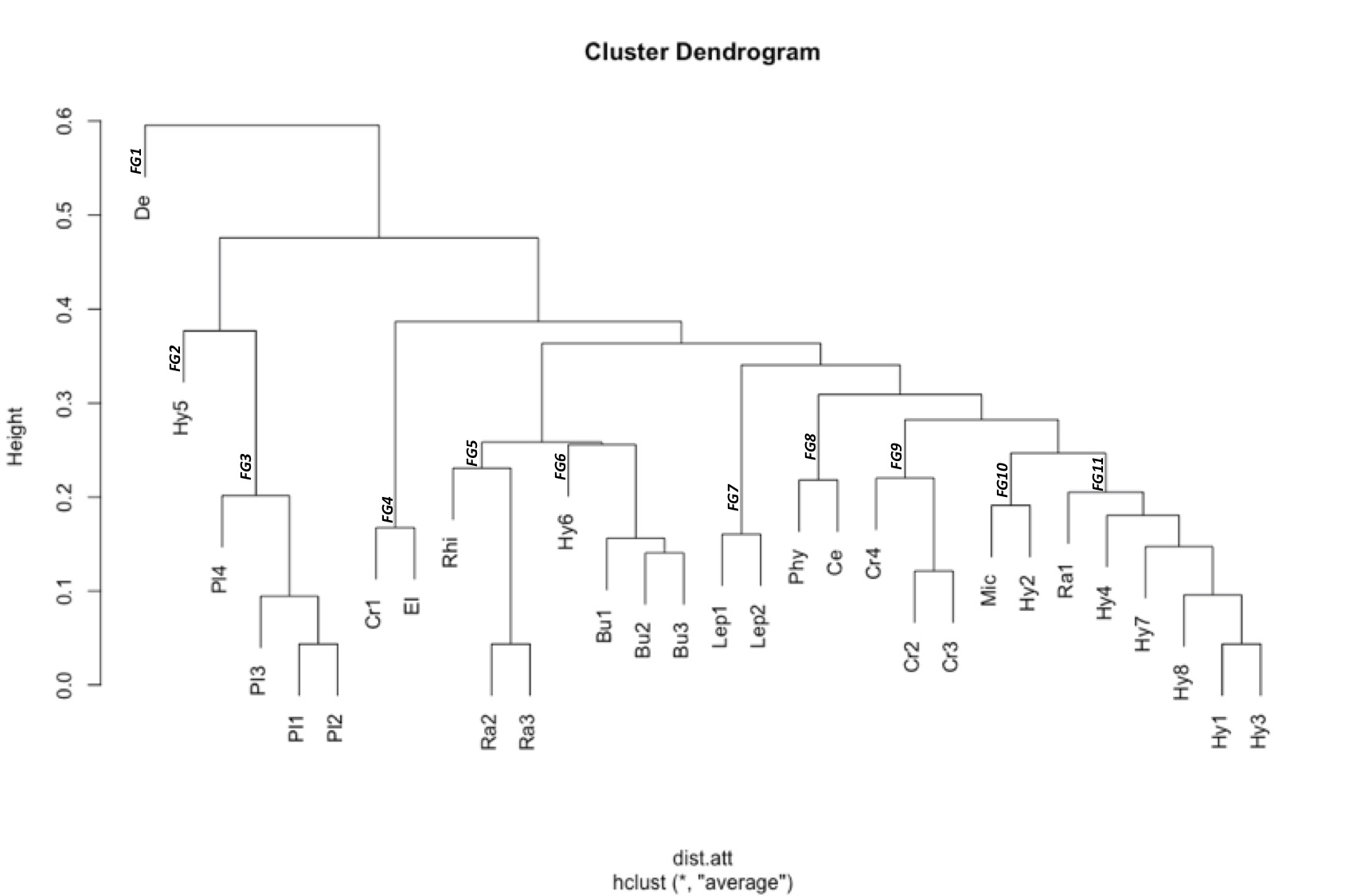
**

**FIGURE S4** Dendrogram of functional groups of Lacandona amphibian species, using Euclidian Distance, tested functional groups by ANOSIM, are indicated by bold-italics in branches.

**References**

Beck, C.W. (1998) Mode of fertilization and parental care in anurans. *Animal Behaviour,* **55,** 439-449.

Campbell, J.A. (1998) *Amphibians and reptiles of northern Guatemala, the Yucatán, and Belize*. University of Oklahoma Press, Norman.

Cortés Gómez, A.M., Ramírez Padilla, M.P. & Urbina Cardona, N. (2015) Protocolo para la medicion de rasgos funcionales en anfibios. *Ecología Funcional como aproximación al estudio, manejo y conservación de la biodiversidad: protocolos y aplicaciones* (ed. B. Salgado Negret), pp. 126-179. Instituto de Investigación de Recursos Biológicos Alexander von Humboldt, Bogotá, Colombia.

Donnelly, M.A. & Guyer, C. (1994) Patterns of reproduction and habitat use in an assemblage of Neotropical hylid frogs. *Oecologia (Berlin),* **98,** 291-302.

Duellman, W.E. & Trueb, L. (1994) *Biology of amphibians*. Johns Hopkins University Press, Baltimore.

Lee, J.C. (2000) *A field guide to the amphibians and reptiles of the Maya world. The lowlands of Mexico, northern Guatemala, and Belize*. Cornell University Press, Ithaca & London.

Olalla-Tarraga, M.A., Diniz-Filho, J.A.F., Bastos, R.P. & Rodriguez, M.A. (2009) Geographic body size gradients in tropical regions: water deficit and anuran body size in the Brazilian Cerrado. *Ecography,* **32,** 581-590.

Pittman, S.E., Jendrek, A.L., Price, S.J. & Dorcas, M.E. (2008) Habitat selection and site fidelity of Cope's gray treefrog (Hyla chrysoscelis) at the aquatic-terrestrial ecotone. *Journal of Herpetology,* **42,** 378-385.

Ribeiro, J., Colli, G., Batista, R. & Soares, A. (2017) Landscape and local correlates with anuran taxonomic, functional and phylogenetic diversity in rice crops. *Landscape Ecology,* **32,** 1599-1612.

Toft, C.A. (1981) Feeding ecology of Panamanian litter anurans: patterns in diet and foraging mode. *Journal of Herpetology,* **15,** 139-144.

Toledo, R.C. & Jared, C. (1993) Cutaneous Adaptations to Water-Balance in Amphibians. *Comparative Biochemistry and Physiology a-Physiology,* **105,** 593-608.

Vitt, L.J. & Caldwell, J.P. (2014) *Herpetology: an introductory biology of amphibians and reptiles. Fourth edition*. Elsevier, Amsterdam, Boston etc.

Wells, K.D. (2007) *The ecology and behavior of amphibians*. University of Chicago Press, Chicago & London.

Zug, G.R., Vitt, L.J. & Caldwell, J.P. (2001) *Herpetology : an introductory biology of amphibians and reptiles,* 2nd edn. Academic Press, San Diego, Calif.
